# Supplementary material for: Development of efficient aqueous organic redox flow batteries using ion-sieving sulfonated polymer membranes
Source: Nat Commun. 2022 Jun 8;13:3184. doi: 10.1038/s41467-022-30943-y (PMC9177609; doi:10.1038/s41467-022-30943-y)
Supplement: Supplementary file 1 — Supplementary information [file 41467_2022_30943_MOESM1_ESM.pdf]

Supplementary Information for

Development of efficient aqueous organic redox flow batteries using ion-sieving sulfonated polymer membranes

Chunchun Ye[1,2,6], Anqi Wang[1,6], Charlotte Breakwell[3], Rui Tan[1], C. Grazia Bezzu[2], Elwin Hunter-Sellars[1], Daryl R. Williams[1], Nigel Brandon[4], Peter A. A. Klusener[5], Anthony R. Kucernak[3], Kim E. Jelfs[3], Neil B. McKeown[2]*, Qilei Song[1]*

[1]Department of Chemical Engineering, Imperial College London, London SW7 2AZ, UK.
[2]EaStCHEM, School of Chemistry, University of Edinburgh, Edinburgh, EH9 3FJ, UK.
[3]Department of Chemistry, Imperial College London, London SW7 2AZ, UK.
[4]Department of Earth Science and Engineering, Imperial College London, London, UK.
[5]Shell Global Solutions International B.V., Shell Technology Centre Amsterdam, Grasweg 31, 1031 HW Amsterdam, The Netherlands.
[6]These authors contributed equally.
*Correspondence to: neil.mckeown@ed.ac.uk and q.song@imperial.ac.uk

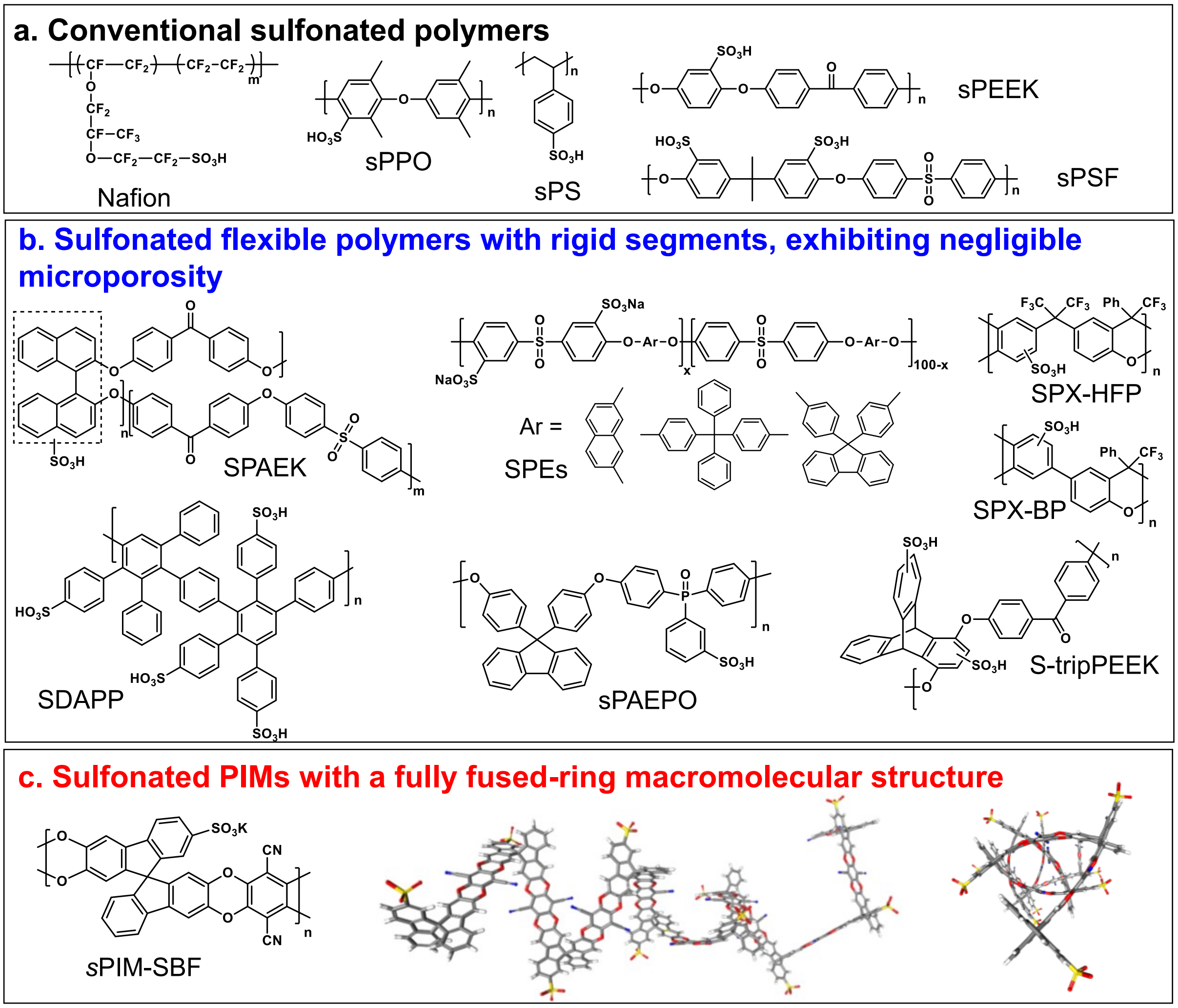

Supplementary Figure 1 | Examples of conventional sulfonated polymers. a, sPPO = sulfonated poly(phenylene Oxide); sPS = Sulfonated polystyrene; sPEEK = sulfonated polyetheretherketone; sPSF = sulfonated poly sulfone. **b,** SPAK= sulfonated poly(arylene ether ketone)[1]; SPEs = Sulfonated Poly(arylene ether sulfone)s[2]; SPX-HFP = sulfonated poly(xanthene hexafluoroisopropylidene)[3]; SPX-BP = sulfonated poly(xanthene dihydroxybiphenyl)[3]; SDAPP = sulfonated Diels–Alder poly(phenylene)[4]; sPAEPO = sulfonated poly(arylene ether phosphine oxide)[5]; S-tripPEEK = sulfonated triptycene poly(ether ketone)[6]; *s*PIM-SBF = sulfonated 9,9'-spirobifluorene based polymer of intrinsic microporosity. **c,** Chemical structure of *s*PIM-SBF (left side), and molecular models from front view (middle side) and side view (right side) of *s*PIM-SBF. *s*PIM-SBF molecular model shows its contorted and rigid shape that generates intrinsic microporosity due to inefficient packing of the polymer chain in the solid state.

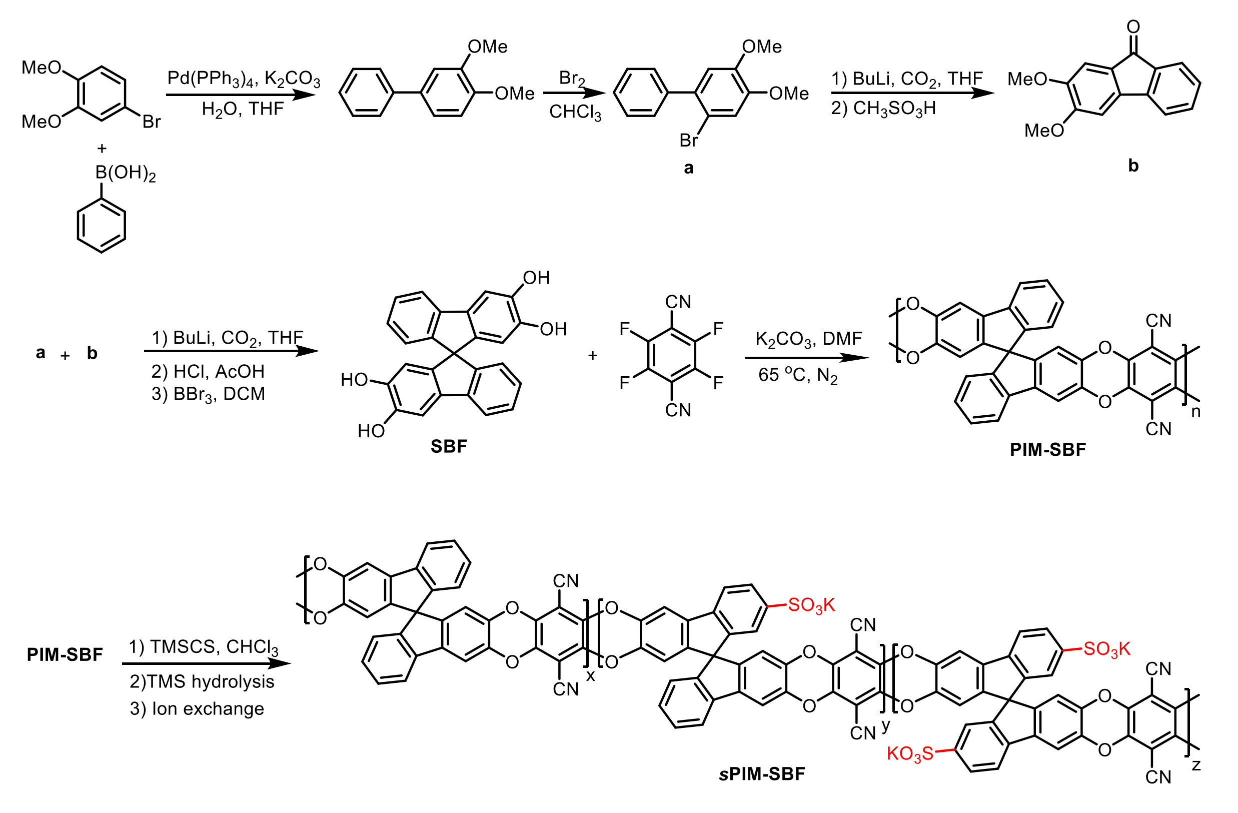

Supplementary Figure 2 | Synthetic route of *s*PIM-SBF polymers.

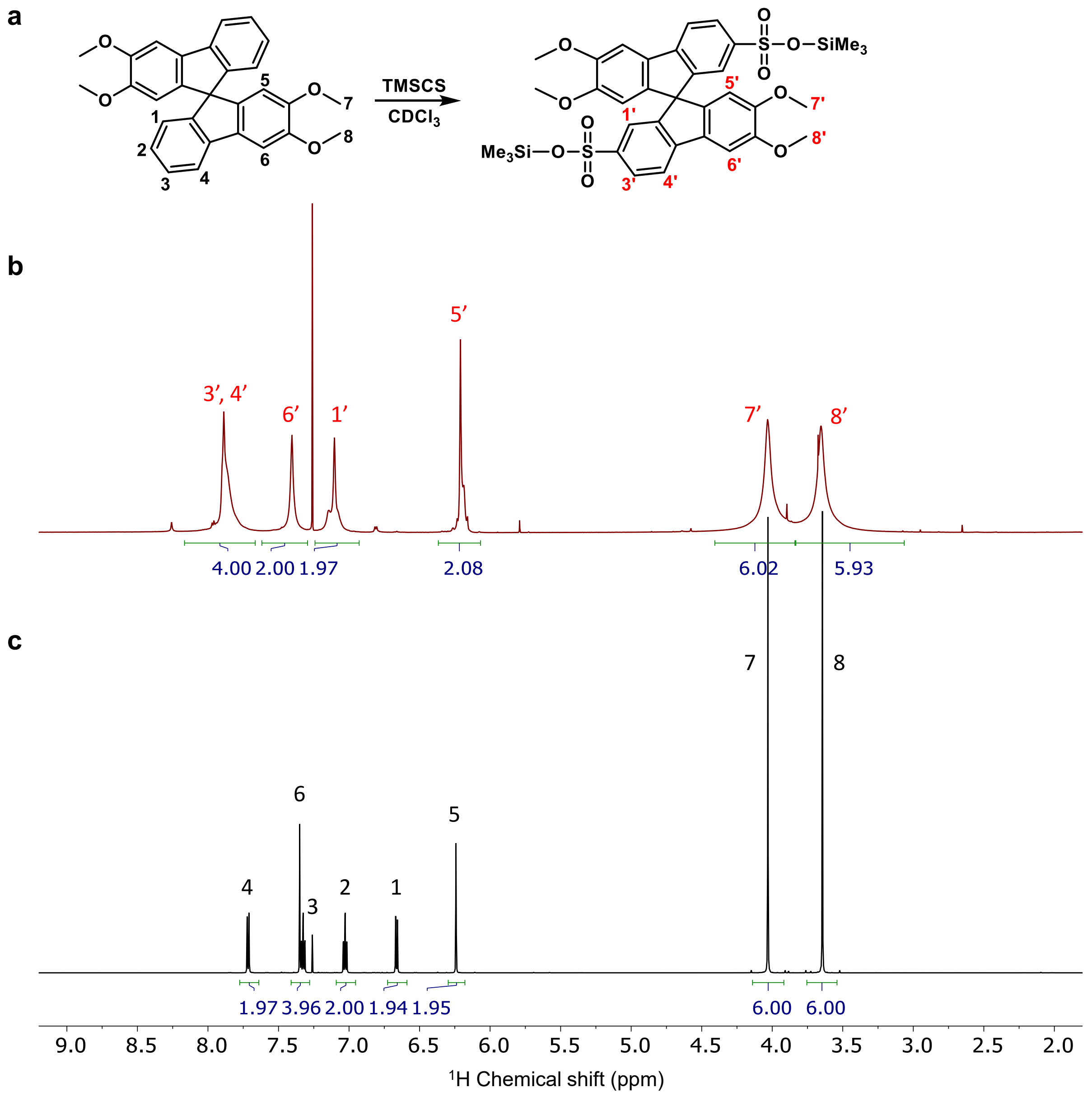

Supplementary Figure 3 | Stacked ^{1}H NMR spectra and peak assignments of model compounds in $CDCl_3$. a, Sulfonation route of model compound using TMSCS as the sulfonation agent in $CDCl_3$. **b,** ^{1}H NMR spectrum of sulfonated SBF monomer precursor 2,2',3,3'-tetramethoxy-6,6'-ditrimethylsiloxysulfonyl-9,9'-spirobifluorene. **c,** ^{1}H NMR spectrum of SBF monomer precursor 2,2',3,3'-tetramethoxy-9,9'-spirobifluorene. ^{1}H NMR analysis confirmed that sulfonate groups were only added to the para site to single phenyl-phenyl bond of the additional fused benzene rings in SBF units.

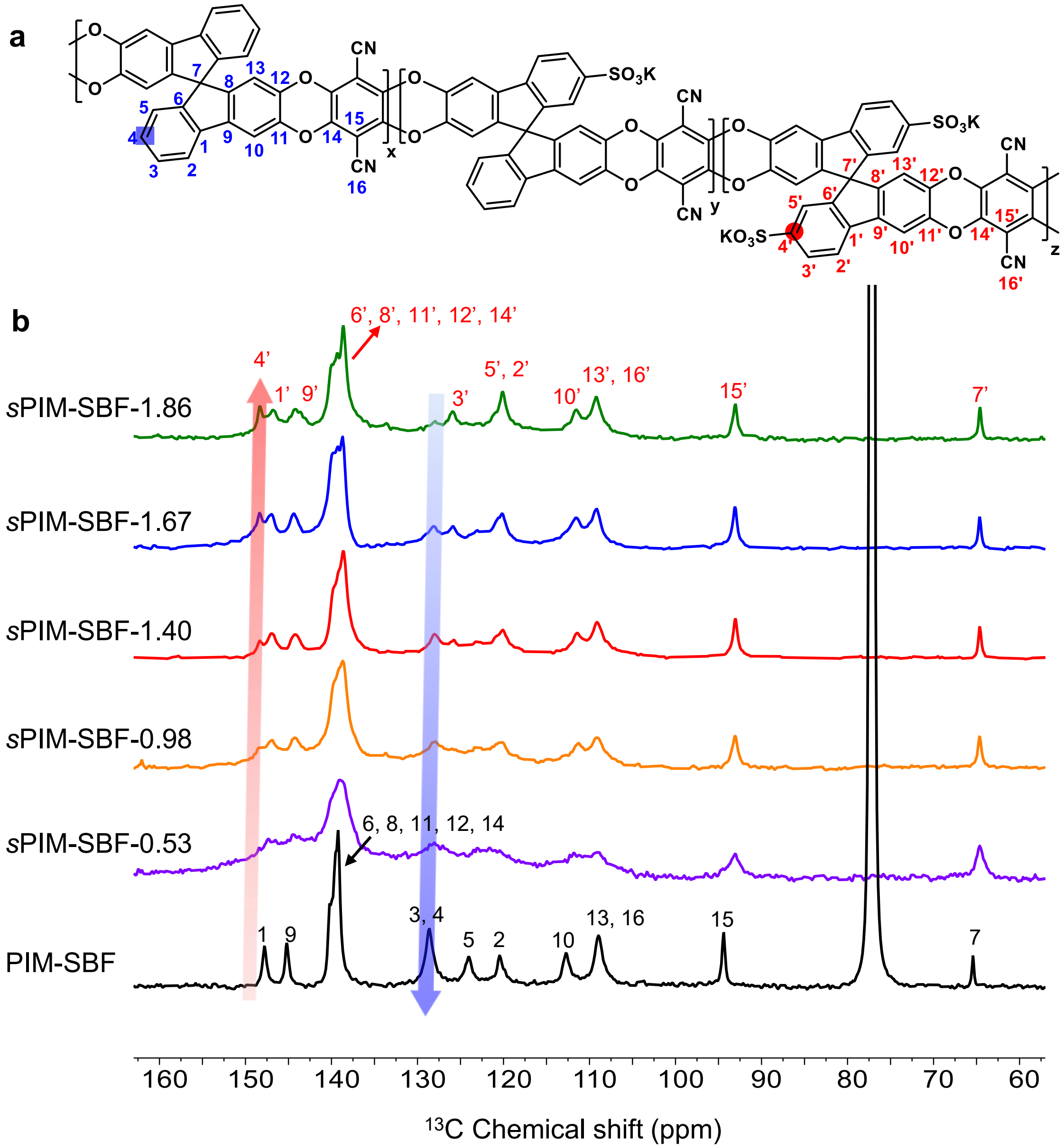

Supplementary Figure 4 | Liquid state ^{13}C NMR spectra and peak assignment of *s*PIM-SBF polymers. a, Chemical structure of *s*PIM-SBF polymers. **b,** Liquid state ^{13}C NMR spectra of PIM-SBF and *s*PIM-SBF polymers with varied degree of sulfonation.The resonance at 144.8 ppm (4') was attributed to carbon environment directly bound to sulfonate groups. Gradually increased resonance at 148.2 ppm (4') and decreased resonance at 128.0 ppm (4) indicated that the degree of sulfonation could be controlled by regulating the molar ratio between TMSCS and PIM-SBF in the reaction[4].

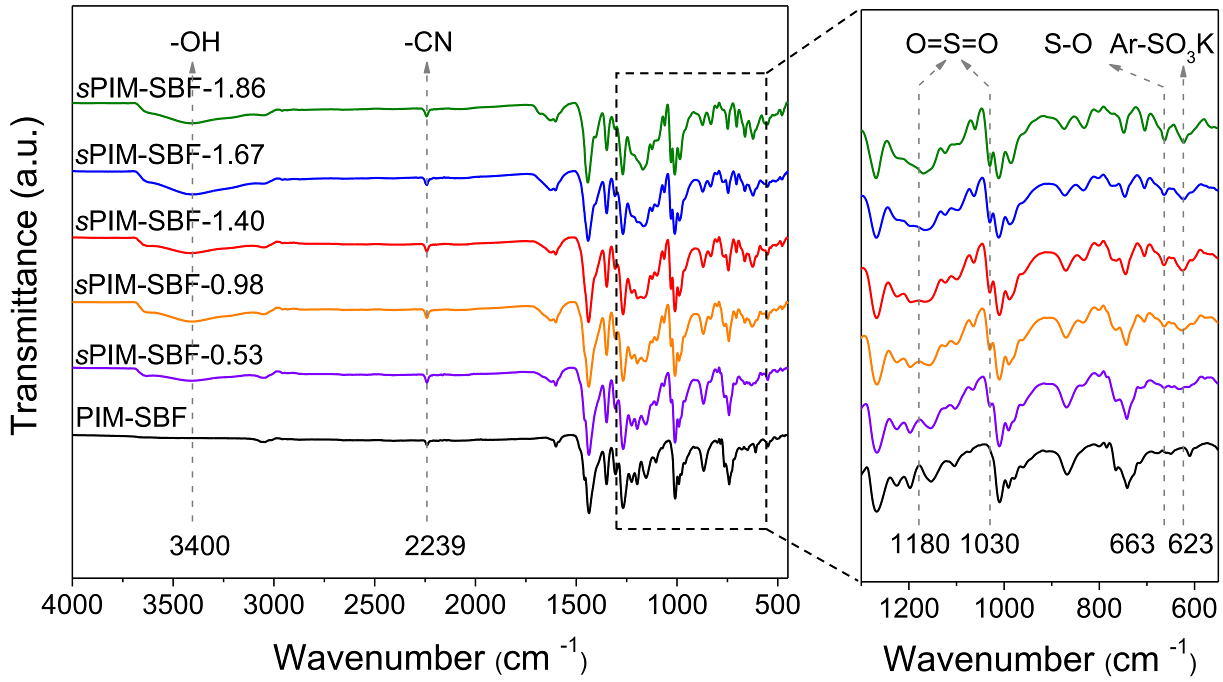

Supplementary Figure 5 | FTIR spectra of *s*PIM-SBF polymers. All structures showed a characteristic stretching peak of the nitrile groups at 2239 cm^{-1}, suggesting no obvious nitrile hydrolysis reaction during the sulfonation[7]. Sharp peaks at 1180 and 1030 cm^{-1} are corresponded to asymmetric and symmetric stretching vibration of S=O in sulfonate groups[6]. Visible increasing absorption intensity for sulfonate groups was also observed with enhancing the degree of sulfonation from *s*PIM-SBF-0.53 to *s*PIM-SBF-1.86.

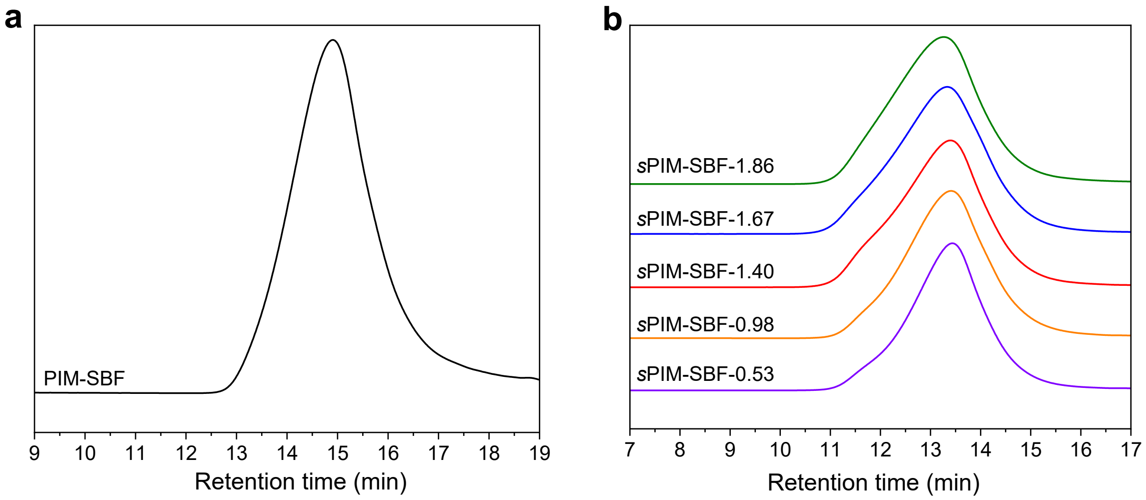

Supplementary Figure 6 | GPC traces of a, original PIM-SBF and b, *s*PIM-SBF polymers. GPC results of original PIM-SBF and *s*PIM-SBF polymers show high molecular weights and relatively narrow molecular weight distributions. These GPC results suggest no or negligible degradation of *s*PIM-SBF polymeric backbones by using TMSCS as the sulfonating agent and performing sulfonation functionalization under mild conditions (Extended data in Supplementary Table 1).

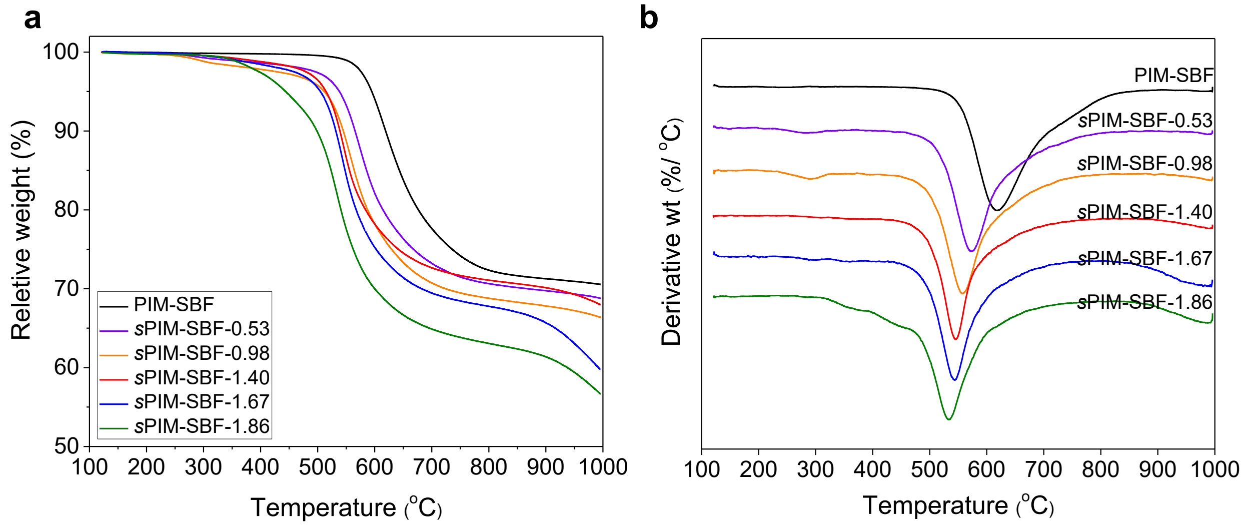

Supplementary Figure 7 | Thermal stability of *s*PIM-SBF polymers. a, TGA thermogram of PIM-SBF and *s*PIM-SBF polymers. **b,** Differential thermo-gravimetric plot of PIM-SBF and *s*PIM-SBF polymers. *s*PIM-SBFs were thermally stable up to 279-356 °C depending on the different degree of sulfonation, and after that they underwent the loss of pendant sulfonate groups[8] (Extended data in Supplementary Table 2).

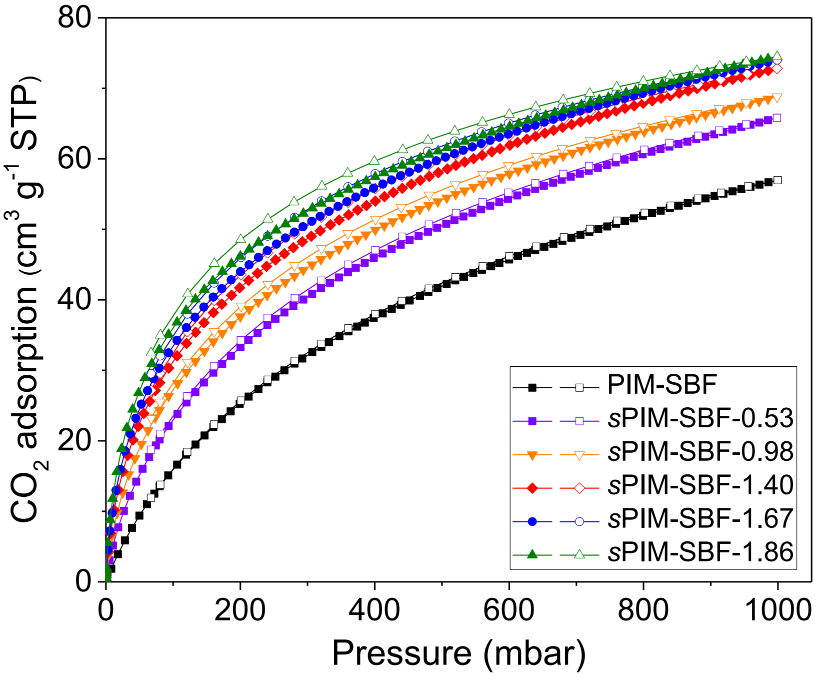

Supplementary Figure 8 | CO_2 sorption isotherms of *s*PIM-SBF polymers. Solid symbols: adsorption; open symbols: desorption.

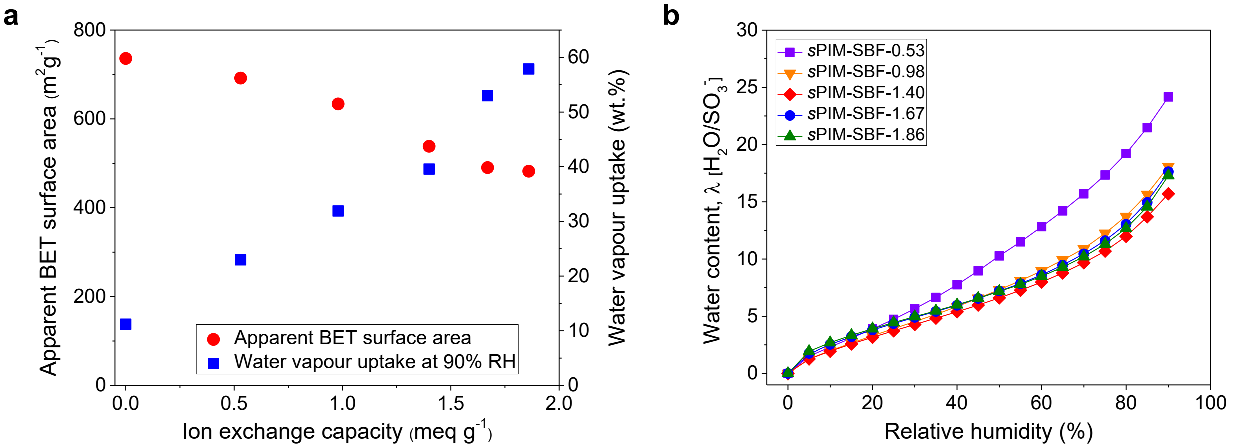

Supplementary Figure 9 | Water uptake of *s*PIM-SBF polymers. a, Apparent BET surface area and water vapour uptake at 90% RH as a function of IEC values for *s*PIM-SBF membranes. In spite of the highest surface area of 773 m^2 g^{-1} and micropore volume of 0.227 cm^3 g^{-1} among all PIM polymers, original PIM-SBF adsorbed only 11.2 wt.% water vapour due to its hydrophobicity. The water vapour uptake for *s*PIM-SBFs increased from 23.0 to 57.9 wt.% on increasing the degree of sulfonation from 0.53 to 1.86 within their backbones, respectively. Therefore, the high water sorption capacity is achieved by the membranes that possess both high microporosity and hydrophilicity. **b,** Water content λ as a function of RH for *s*PIM-SBF membranes measured at 25 °C. The change of sorption curves as a function of sulfonation degree was not pronounced, with the exception of *s*PIM-SBF-0.53 membrane indicating a morphology difference at a low degree of sulfonation. In these typical nonlinear sorption isotherms, the first steady ramp resulted from the initial solvation of sulfonate groups by adsorption of strongly bound water up to 60% RH. Subsequently, a percolated water network of nanodomains was formed by continuous swelling of more mobile water molecules at higher RH[9] (Extended data in Supplementary Table 3).

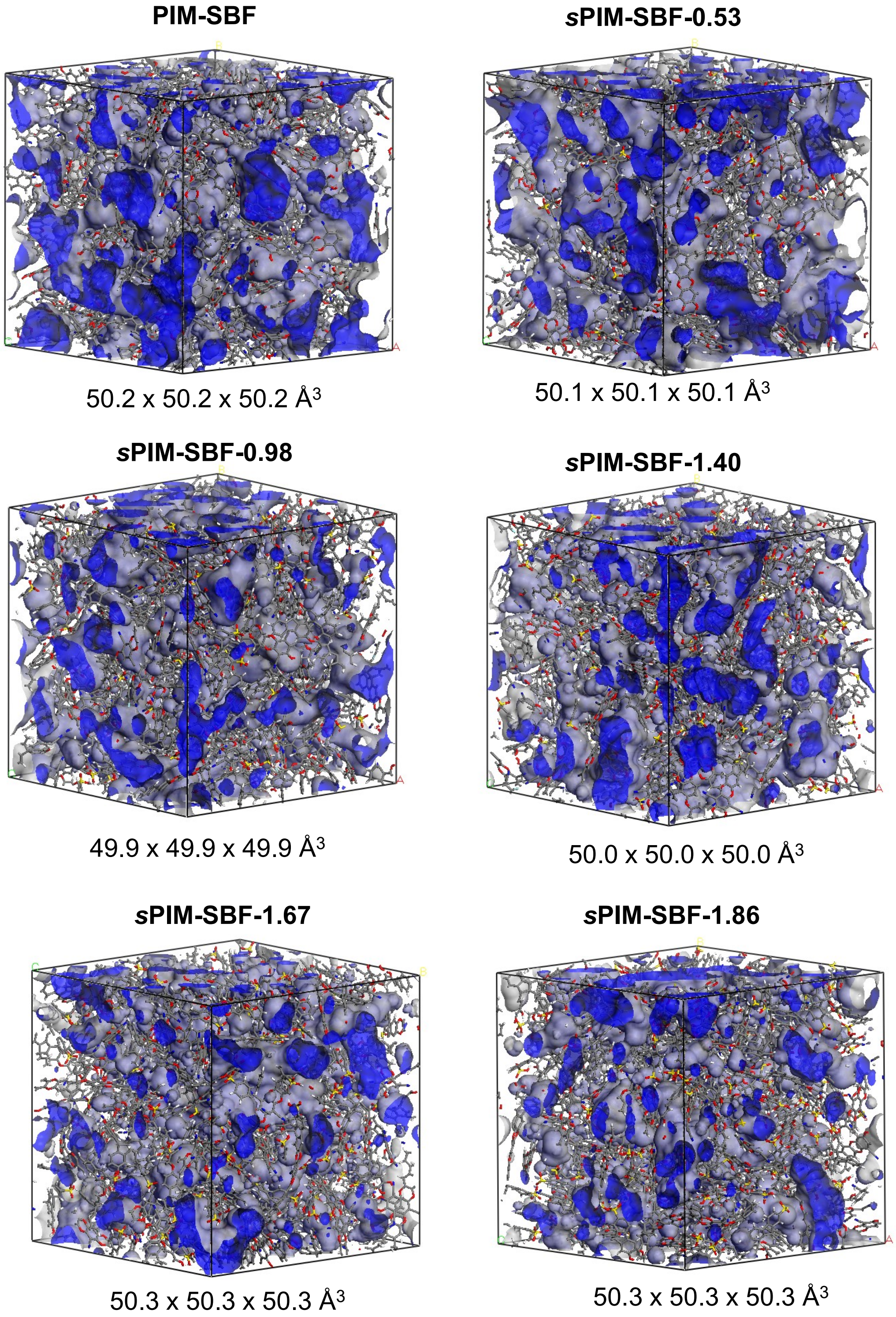

Supplementary Figure 10 | Snapshots of chain packing simulations for PIM-SBF and *s*PIM-SBFs visualised in Material Studio. Connolly surface for a probe radius of 1.82 Å (*i.e.,* N_2-sized probe) is highlighted in blue, with free volume highlighted in grey.

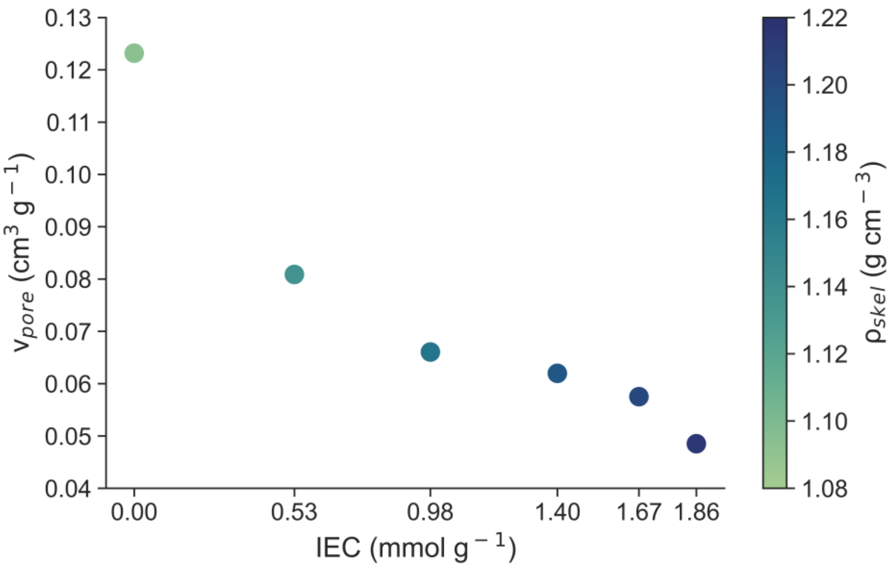

Supplementary Figure 11 | Accessible pore volumes of the simulated PIM-SBF and *s*PIM-SBF models with respect to a He size probe (radius = 1.3 Å). A heat map is used to highlight the simulated skeletal densities (ρ_{skel}).

For direct comparison with experimental measurements, skeletal densities (ρ_{skel}) were derived from the geometric pore volumes (v_{pore}) and final bulk densities (ρ_{sim}) of the computational models (Equation: $\frac{1}{\rho_{skel}} = \frac{1}{\rho_{sim}} - \frac{v_{pore}}{m}$). A helium sized probe (radius = 1.3 Å) was used here to mirror the skeletal densities measured experimentally by helium pyconometry. Simulated skeletal densities showed some deviation from those obtained experimentally. Comparison of simulated and experimental densities becomes difficult for polymers with large pore volumes, such as PIM-SBF, since the fixed framework assumed by the geometric pore volume measurements cannot compare directly with experimental helium pyconometry, where large pore volumes are exaggerated by host-guest interactions. This observation has been reported previously for simulations of PIM-1, where disparities (~8%) were observed between experimental and simulated skeletal densities[10]. Nonetheless, good agreement is observed between the trends of simulated and experimental skeletal densities. Overall, the simulations reveal a decrease in pore volume as well as an increase in skeletal density for *s*PIM-SBFs with greater IEC, suggesting that sulfonation has a direct influence on the size and shape of the microporous network (Extended data in Supplementary Table 5).

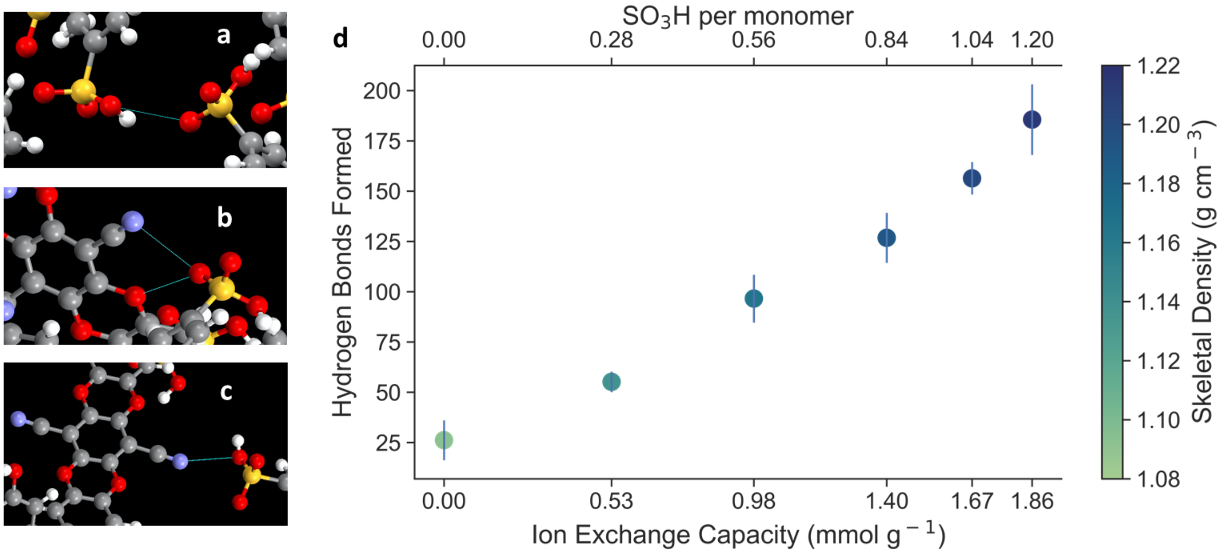

Supplementary Figure 12 | Hydrogen bonds of the *s*PIM-SBF polymers. a-c, Hydrogen bonds between sulfonic acid groups and neighbouring electronegative atoms along the polymer backbone. Carbon, oxygen, nitrogen, sulphur and hydrogen atoms are coloured as grey, red, purple, yellow and white respectively. **d,** A scatter graph of the number of hydrogen bonds formed with respect to IEC and level of sulfonation. The number of hydrogen bonds is the weighted average across the five independent models, with error bars shown to indicate standard deviation. A heat map is used to compare the increasing hydrogen bonds to simulated skeletal densities. Hydrogen bonding in the polymer networks was analysed in Mercury[11]. Visualisation reveals that a hydrogen bonding network is formed throughout the polymer by the introduction of sulfonic acid pendant groups. Hydrogen bonds are formed between the oxygen atoms of the sulfonic acid groups and either neighbouring nitrogen/oxygen atoms along the polymer backbone or adjacent sulfonic acid pendant groups (Supplementary Figure 12 a-c). The addition of these strong inter and intra molecular forces throughout the material causes the polymer chains to pack more closely, leading to the increased skeletal densities and reduction in free volumes and pore apertures that have been observed for the dehydrated *s*PIM-SBF series. As shown in Supplementary Figure 12d, the number of hydrogen bonds present in the network increases exponentially with increasing IEC. Although this hydrogen bonding network will change drastically under aqueous conditions, the increased tendency towards hydrogen bond formation of *s*PIM-SBF polymers with a higher degree of sulfonation may contribute to the increased water uptake that has been observed experimentally for materials with greater IEC.

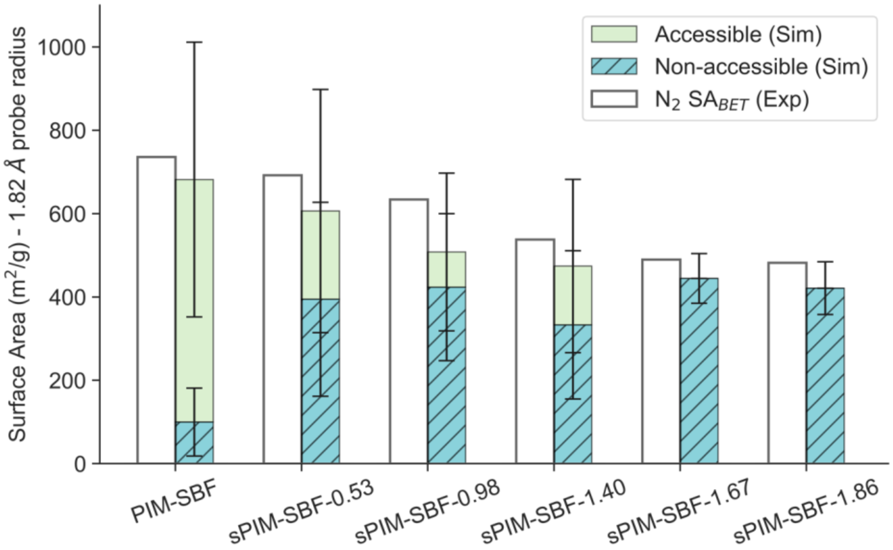

Supplementary Figure 13 | Geometric accessible and non-accessible surface areas with respect to a nitrogen sized probe (radius = 1.82 Å) of the PIM-SBF and *s*PIM-SBF systems. Error bars are indicative of the standard deviation across the 5 independent models. Experimental BET surfaces areas by nitrogen adsorption are also reported for comparison. The geometric surface areas are split into two categories: accessible and non-accessible surface areas (ASA and NASA respectively). ASA is representative of the interconnected free volume in a network, whilst NASA describes any non-connected or isolated voids throughout the network which could become accessible with swelling. The total geometric surface areas with respect to a nitrogen sized probe show excellent agreement with the trends of BET surfaces areas measured experimentally.

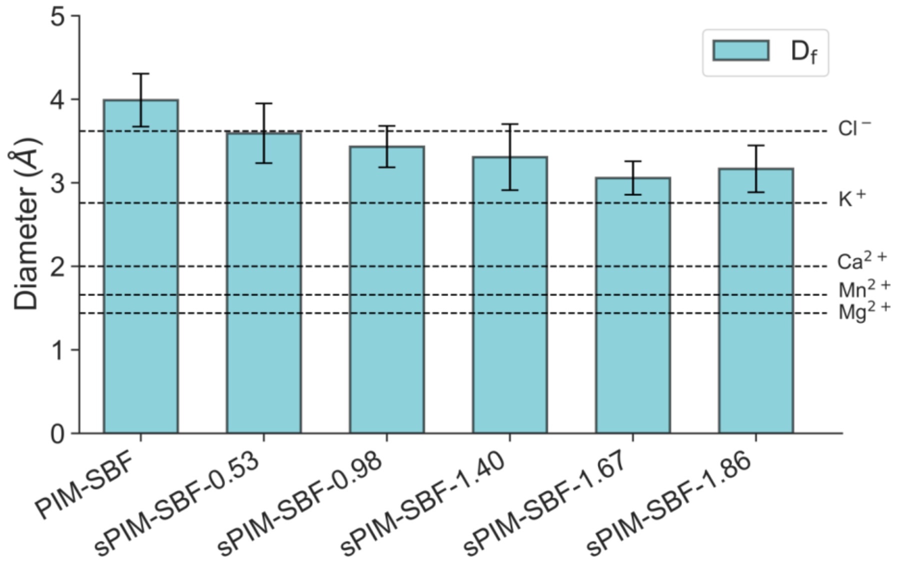

Supplementary Figure 14 | Calculated largest free sphere path (D_f) for the dry-state PIM-SBF and *s*PIM-SBF models with varying degree of sulfonation. Error bars show the standard deviation across the five models for each system. Diameters of some common metal ion are also compared; Mg^{2+} (1.44 Å), Mn^{2+} (1.66 Å), Ca^{2+} (2.00 Å), K^+ (2.76 Å) and Cl^- (3.62 Å). Measurements of the largest cavity diameter (D_i) and largest free sphere path (D_f) for the PIM-SBF and *s*PIM-SBF models are reported in Supplementary Table 6. In particular, D_f defines the largest probe size that can diffuse across the model through interconnected pores. D_f values are compared to the diameters of some common metal ions[12]. As shown in Supplementary Figure 12, the *s*PIM-SBF series are porous to many small metal cations and are therefore suitable candidates for ion exchange membrane applications. Furthermore, the models reveal narrow D_f values (> 3.6 Å) for all of the *s*PIM-SBFs, suggesting that they could all potentially block (or at least hinder) the permeation of large organic molecules.

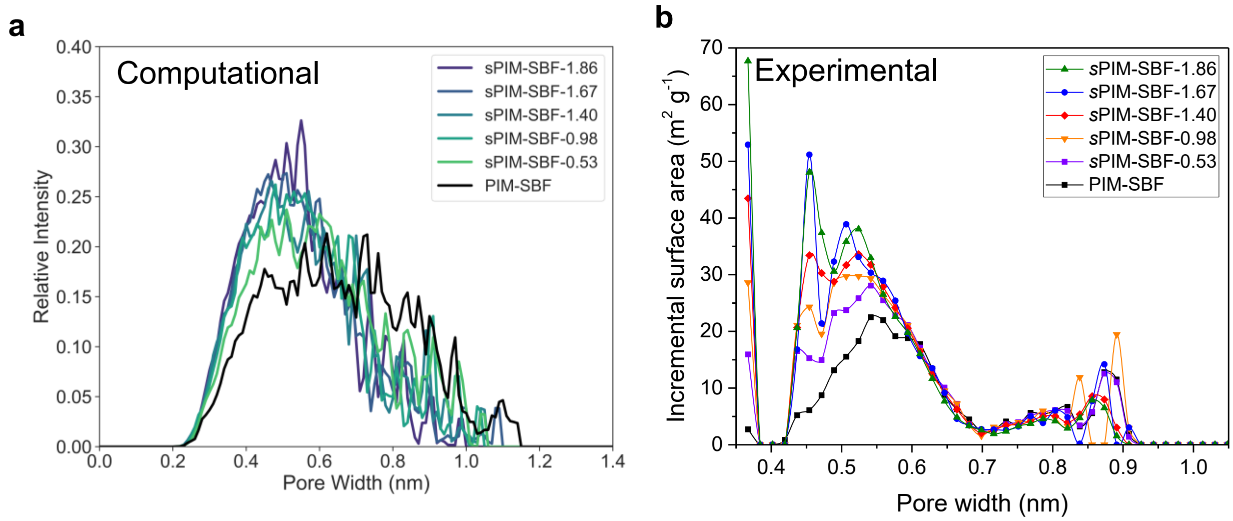

Supplementary Figure 15 | pore size distributions of PIM-SBF and *s*PIM-SBF polymers. a, Geometric pore size distributions of PIM-SBF and *s*PIM-SBF polymers measured from simulations (probe radius = 1.0 Å). Distributions for each system are an average weighting of five individual models. **b,** Experimental pore size distributions of PIM-SBF and *s*PIM-SBF polymers measured by CO_2 sorption and DFT. Qualitatively, the pore size distributions of the simulations are in good agreement with the experimental materials, where PIM-SBF exhibits a broad distribution centred around 0.7 nm, shifting to narrower distributions of smaller micropores with increasing level of sulfonation, centred around 0.5 nm for *s*PIM-SBF-1.86. All of the PIM-SBFs can be considered as microporous materials (pore cavities < 2nm)[13].

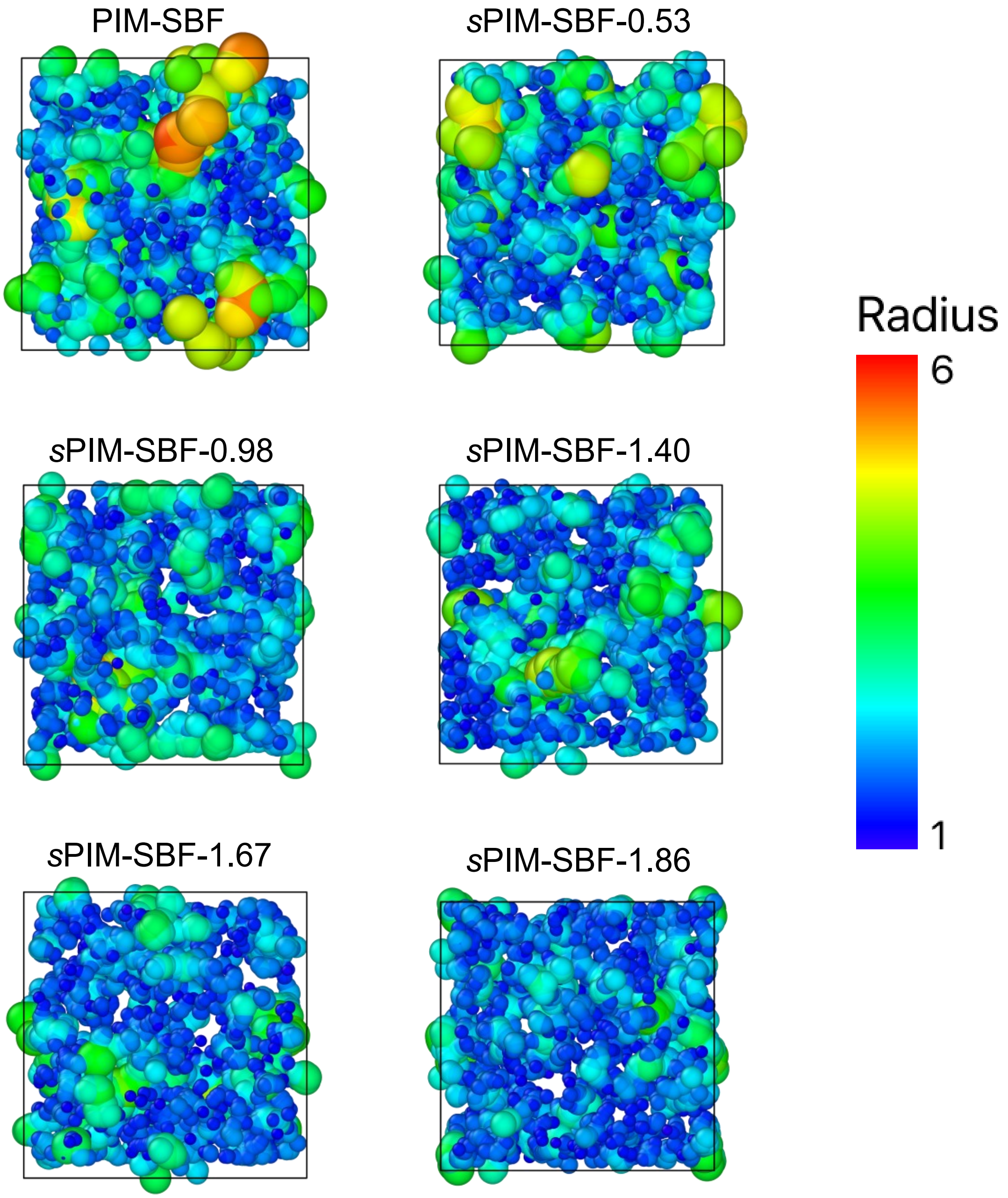

Supplementary Figure 16 | Visual pore size distributions of the *s*PIM-SBF models at various IECs. one out of five of the independent models for each system are shown. Pores are colour coded by their size in Angstrom. Only pores greater than 1.0 Å are shown.

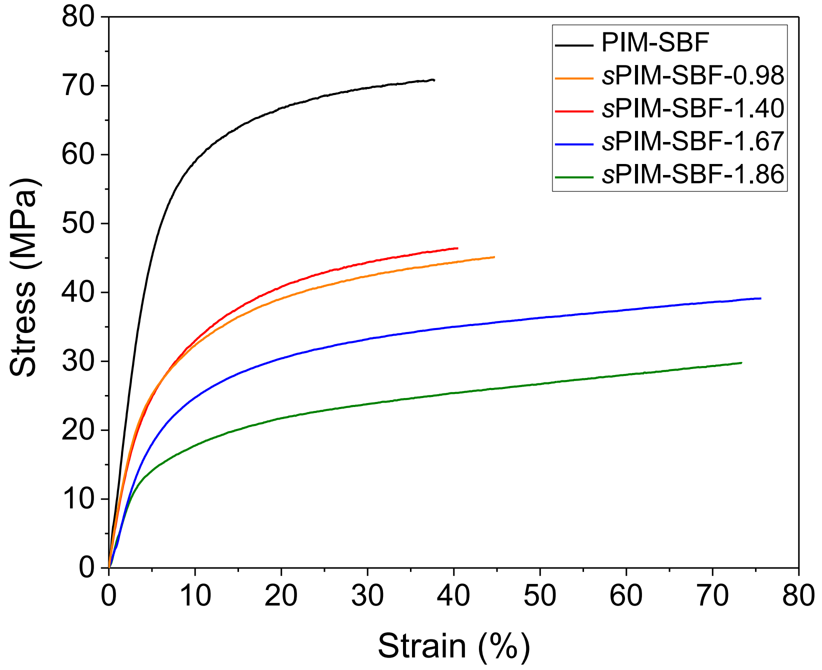

Supplementary Figure 17 | Stress-strain curves of *s*PIM-SBF polymers. *s*PIM-SBF membranes with varied degree of sulfonation displayed adequate mechanical robustness, with ultimate tensile strengths in the range of 29.8-45.3 MPa, and Young's modulus in the range of 0.56-0.89 GPa, respectively (Extended data in Supplementary Table 7).

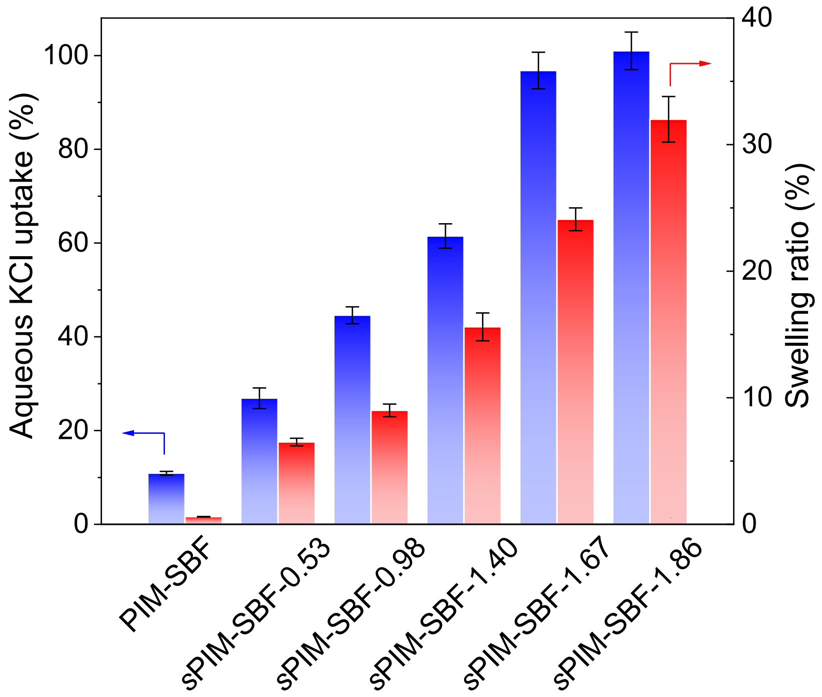

Supplementary Figure 18 | Aqueous KCl uptake and linear swelling ratio of *s*PIM-SBF membranes. Error bars are standard deviations derived from three KCl uptake and swelling ratio measurements based on three different membrane samples, respectively (Extended data in Supplementary Table 3). PIM-SBF membrane showed a relatively low electrolyte uptake of ~ 11% at room temperature, while *s*PIM-SBF membranes exhibited increased electrolyte uptakes from 27% to 101% with increasing degree of sulfonation, showing the similar trend with DVS results. Importantly, *s*PIM-SBF membranes showed low linear swelling ratio (6.5-32.0%), suggesting structural resistance to pore dimension expansion upon hydration, hence maintaining the mechanical robustness of membranes in an aqueous system.

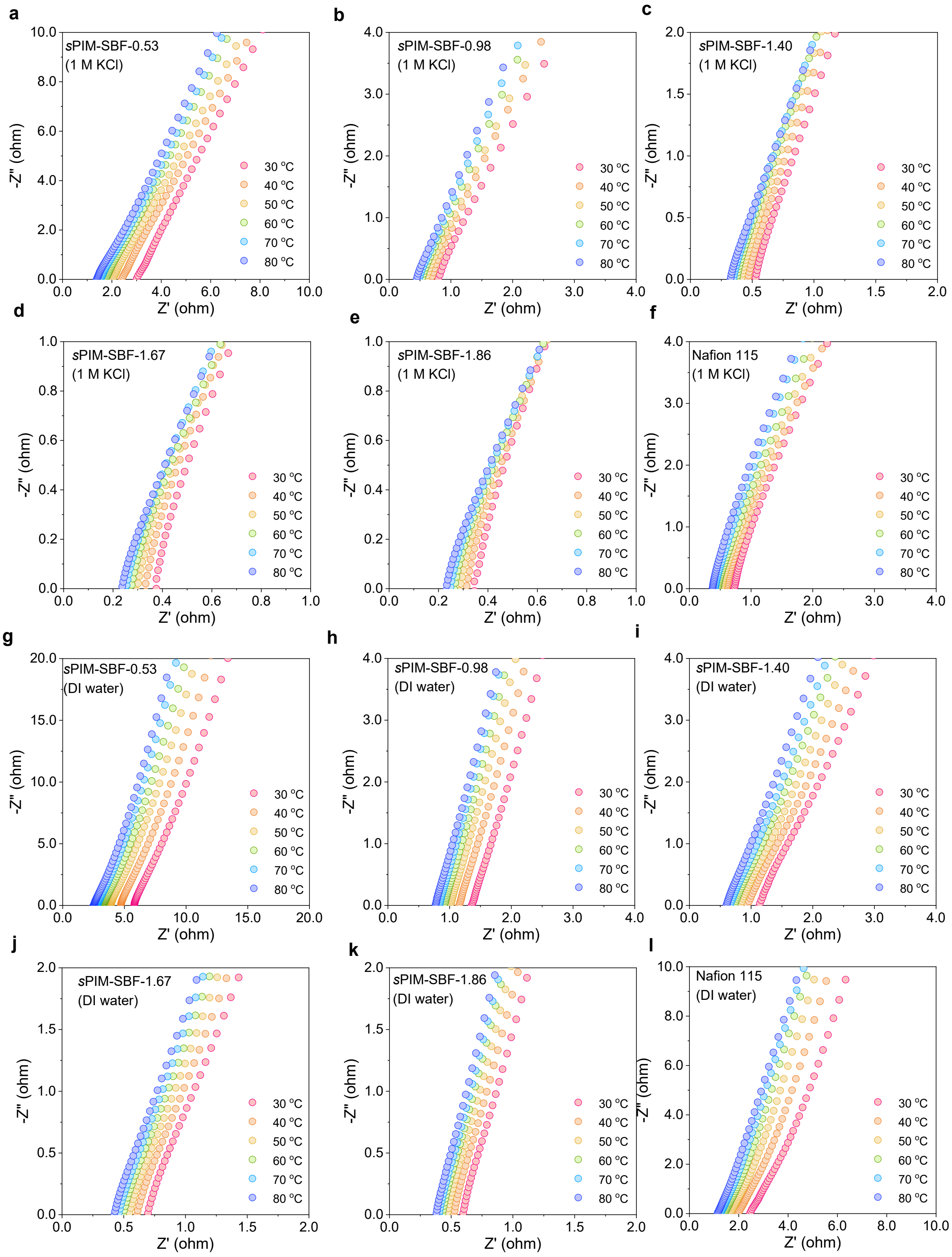

Supplementary Figure 19 | EIS spectra of *s*PIM-SBF and Nafion 115 membranes. a-f, Nyquist plots of *s*PIM-SBF and Nafion 115 membranes measured in 1 M aqueous KCl by EIS in the temperature range of 30-80 °C. **g-l,** Nyquist plots of *s*PIM-SBF and Nafion 115 membranes measured in deionised water by EIS in the temperature range of 30-80 °C.

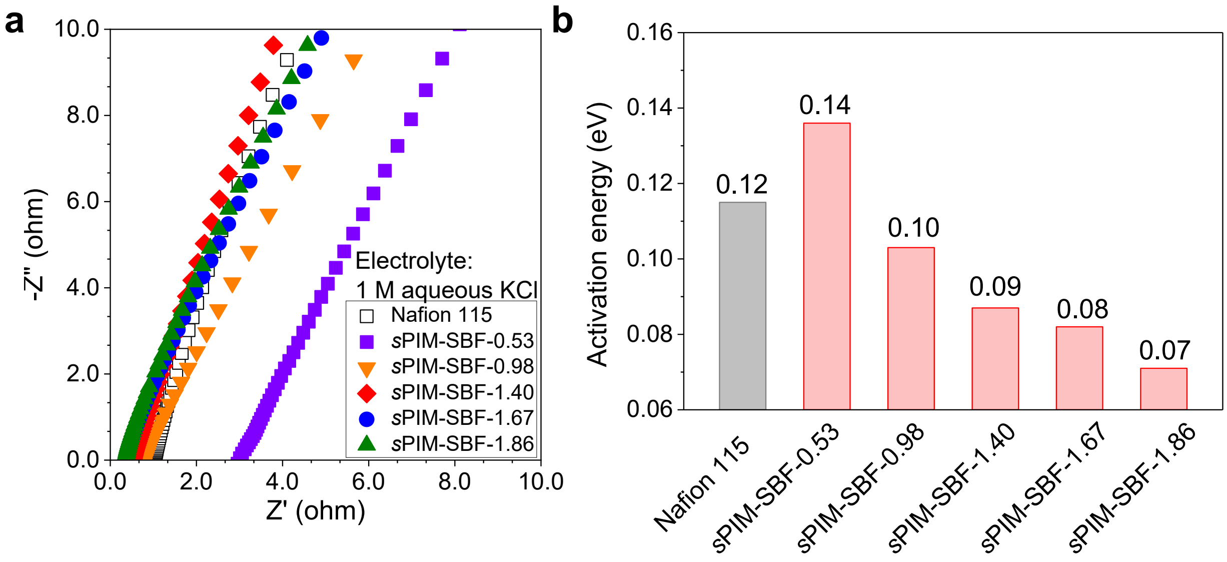

Supplementary Figure 20 | Apparent ionic conductivity and activation energy of *s*PIM-SBF and Nafion 115 membrane. a, Nyqiust plots of *s*PIM-SBF and Nafion 115 membranes measured by EIS in 1 M aqueous KCl at 30 °C. **b,** Activation energy of *s*PIM-SBF and Nafion 115 membranes measured in 1 M aqueous KCl.

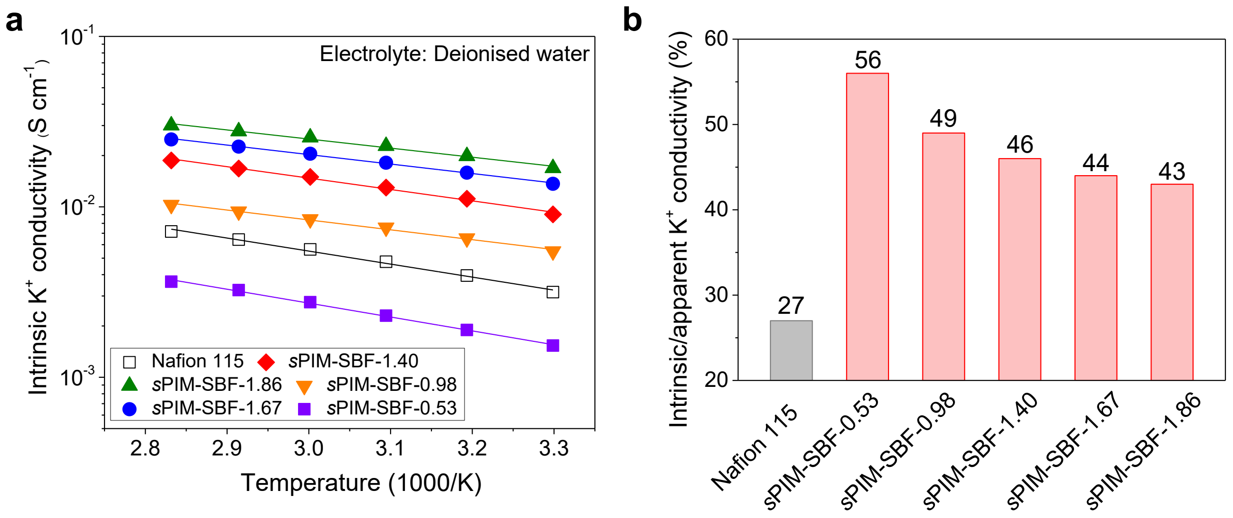

Supplementary Figure 21 | Intrinsic ionic conductivity of *s*PIM-SBF and Nafion 115 membranes. a, Temperature dependence of intrinsic K^+ ionic conductivity of *s*PIM-SBF and Nafion 115 membranes measured in deionised water in the range of 30-80 °C. **b,** The ratio of intrinsic and apparent K^+ conductivity of *s*PIM-SBF and Nafion 115 membranes.

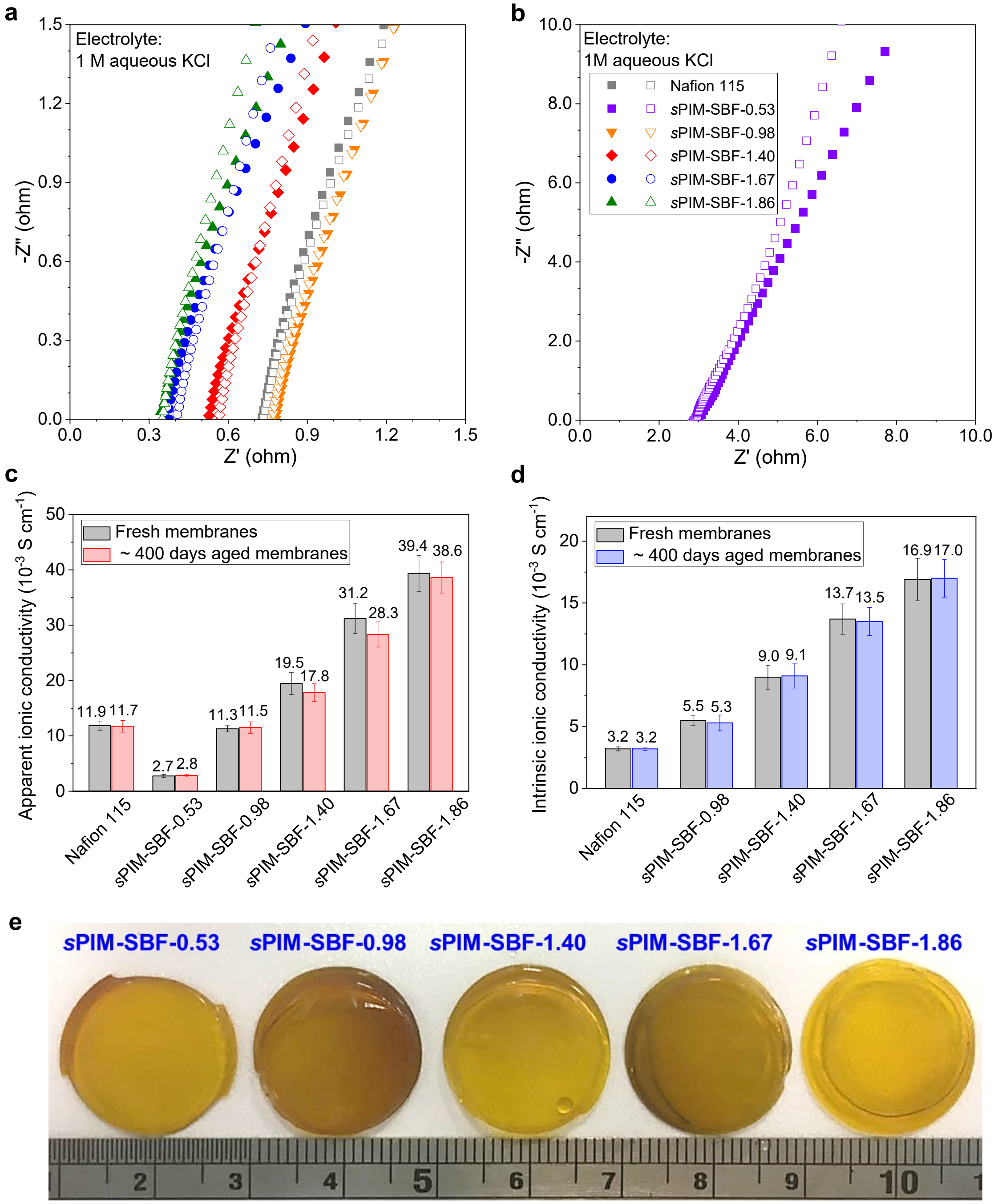

Supplementary Figure 22 | Ionic conductivity evolution of *s*PIM-SBF membranes measured by EIS. a-b, Nyquist plots of fresh and ~400-day-aged *s*PIM-SBF and Nafion 115 membranes measured in 1 M aqueous KCl by EIS at 30 °C. Comparison of **c**, Apparent K^+ ionic conductivity and **d,** Intrinsic K^+ ionic conductivity values of fresh and ~400-day-aged *s*PIM-SBF and Nafion 115 membranes (Extended data in Supplementary Table 8). Error bars are standard deviations derived from three ionic conductivity measurements based on three different samples. The intrinsic ionic conductivity of *s*PIM-SBF-0.53 is not shown due the missing of this membrane. **e,** Photographs of *s*PIM-SBF membranes disassembled from coin cells after ~400 day's aging. Physical aging is a general behaviour of glassy polymers including PIMs and leads to the loss of

free volume and deterioration of membrane performance in the field of gas separation. PIM membranes are generally soaked into methanol (or ethanol) to the swollen state in order to remove any residual casting solvent but also introduce additional free volume upon the evaporation of methanol[14]. In aqueous redox flow battery, however, *s*PIM-SBF membranes are exposed to aqueous electrolytes and keep hydrated and swollen states, which are expected to maintain their microporosity hence ionic transport property. The aging behavior of the swollen *s*PIM-SBF membranes was studied by remeasuring the ionic conductivity after over 400 days' aging. As shown in Supplementary Figure 22 a-d, all *s*PIM-SBF membranes showed no obvious change of the ionic conductivity, and maintained defect-free when disassembled from coin cells over 400 days (Supplementary Figure 22e). The highly stable ionic conductivity of *s*PIM-SBF membranes indicate their stability in battery electrolytes but also minimum the influence on battery performance decay.

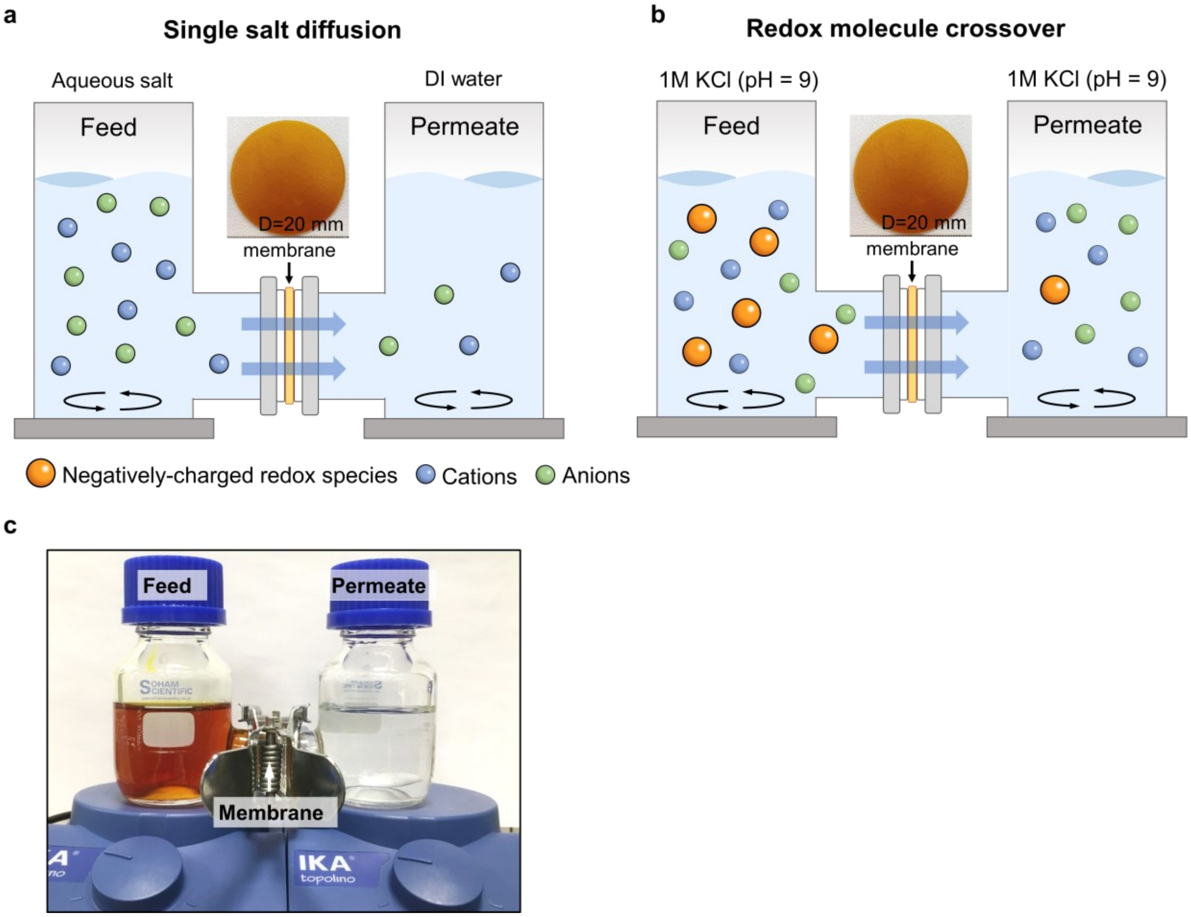

Supplementary Figure 23 | Experimental approach of H-shaped cells for concentration-driven ion diffusion dialysis and redox molecule crossover measurements. Schematics of the H-cell showing **a,** Single salt diffusion dialysis tests and **b,** Redox molecule crossover tests. **c,** Photograph of a typical H-shaped cell.

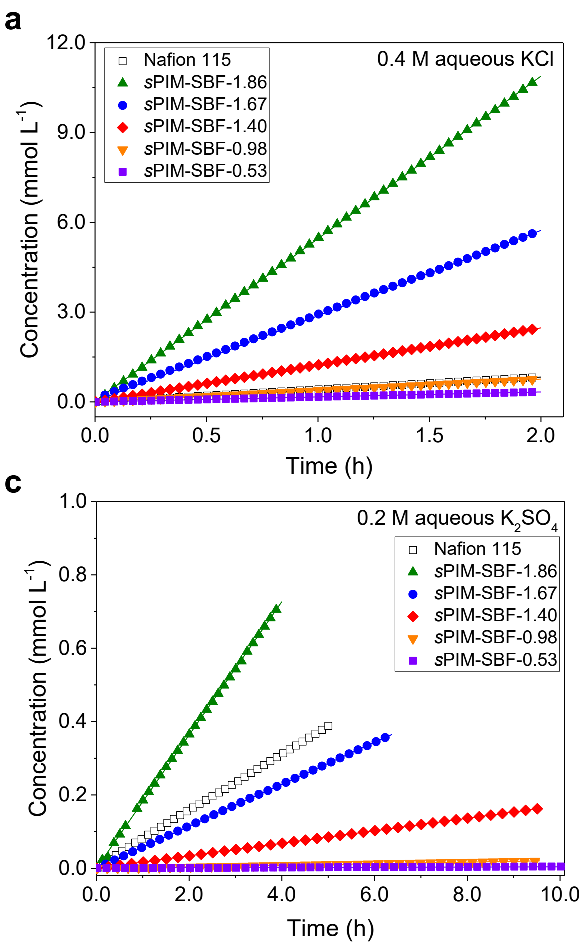

Supplementary Figure 24 | Ion permeation through *s*PIM-SBF and Nafion 115 membranes using concentration-driven dialysis diffusion H-cells. Concentration change as a function of time of **a,** KCl, **b,** KNO_3, **c,** K_2SO_4, and **d,** K_2CO_3 in the permeate side of H-cells assembled with *s*PIM-SBF or Nafion 115 membranes. The feed solution is 0.4 M KCl, 0.4 M KNO_3, 0.2 M K_2SO_4, or 0.2 M K_2CO_3 aqueous solution respectively and the permeate side is deionised H_2O. The temperature for ion permeation measurements is around 25 °C (Extended data in Supplementary Table 9).

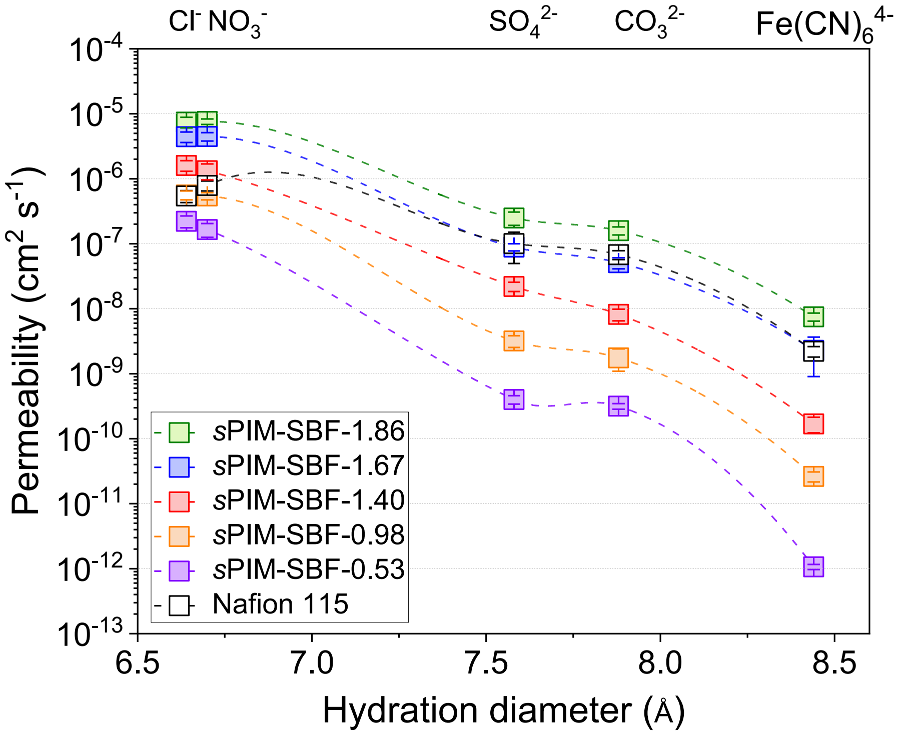

Supplementary Figure 25 | Selective ion permeation of common salts through *s*PIM-SBF and Nafion 115 membranes with thickness of around 150 μm. The feed solution is 0.4 M KCl, 0.4 M KNO_3, 0.2 M K_2SO_4, 0.2 M K_2CO_3 or 0.1 M $K_4Fe(CN)_6$ aqueous solution with varied hydrated diameters of anions (Cl^- = 6.64 Å, NO_3^- = 6.70 Å, SO_4^{2-} = 7.58 Å, CO_3^{2-} = 7.88 Å, $Fe(CN)_6^{4-}$ = 8.44 Å[15]), respectively, and the permeate side is deionised H_2O. The temperature for ion permeation measurements is around 25 °C. Dash lines are added as guides to the eye (Extended data in Supplementary Table 9).

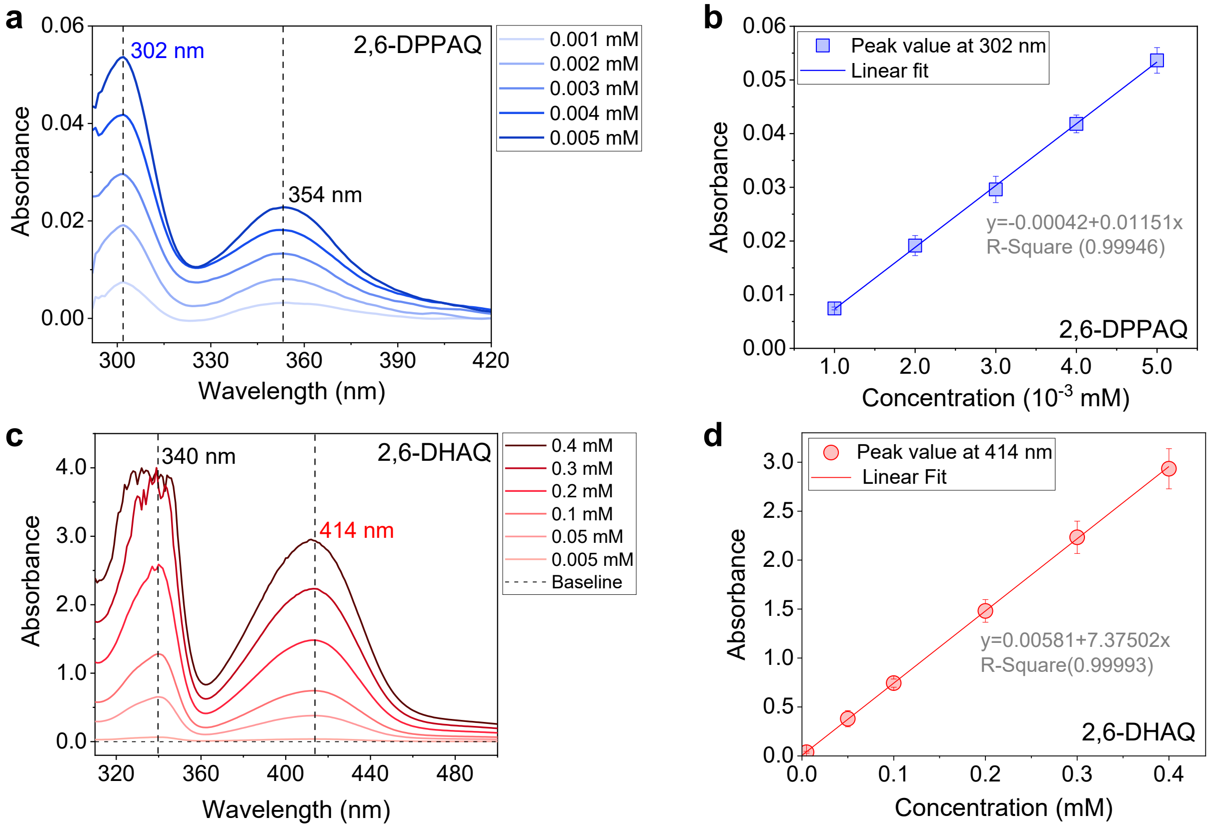

Supplementary Figure 26 | UV-Vis spectra and calibration curves of a&b, 2,6-DPPAQ and c&d, 2,6-DHAQ redox active molecules. The peak absorbance at the wavelength of 302 nm and 414nm was used to calculate the concentration of 2,6-DPPAQ and 2,6-DHAQ molecules, respectively. Error bars in b and d are standard deviations derived from at least three measurements based on three different specimens.

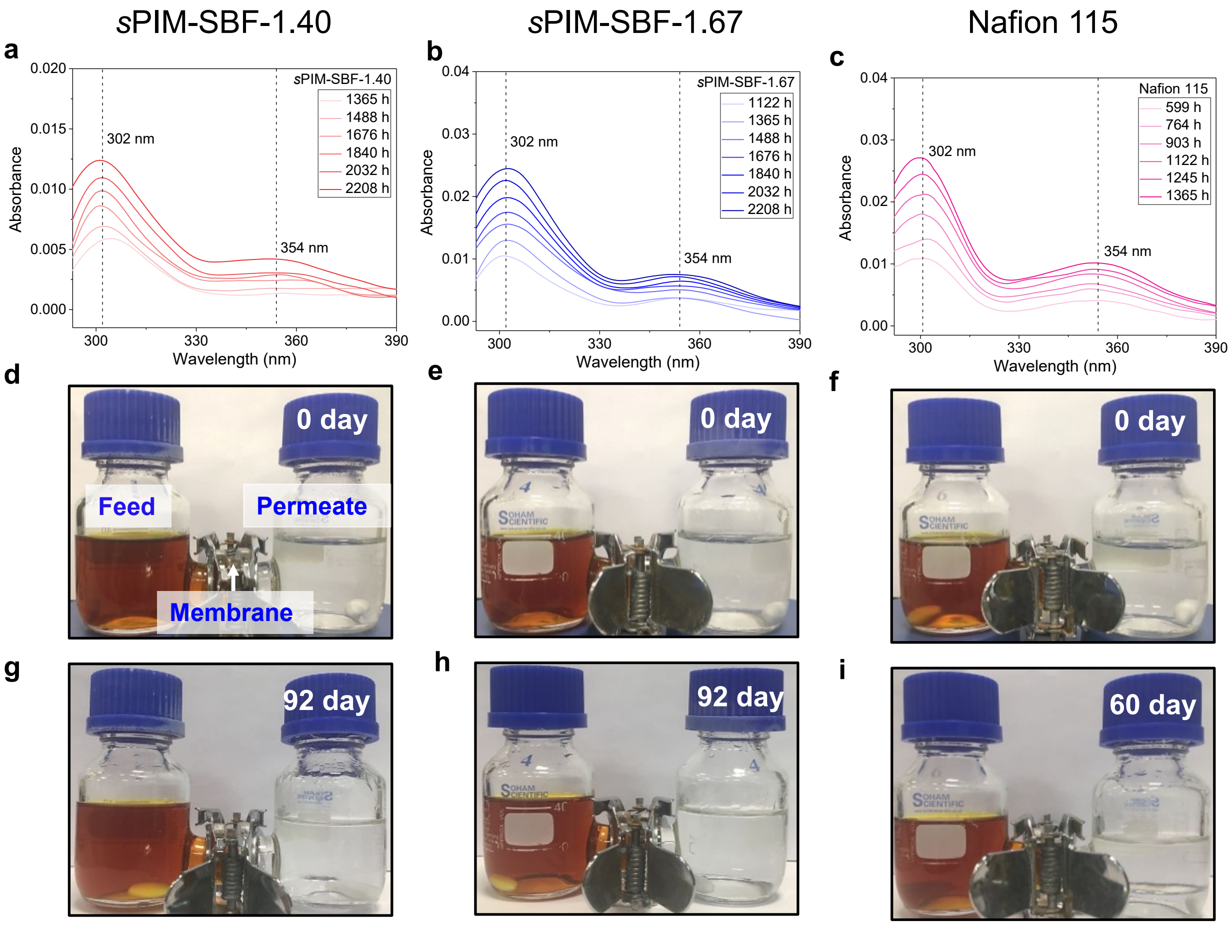

Supplementary Figure 27 | 2,6-DPPAQ crossover through *s*PIM-SBF and Nafion 115 membranes using concentration-driven dialysis diffusion H-cells. UV-Vis spectra of 2,6-DPPAQ as a function of time in the permeate side in H-cells assembled with **a,** *s*PIM-SBF-1.40, **b,** *s*PIM-SBF-1.67, and **c,** Nafion 115 membranes. The feed solution is 0.1 M 2,6-DPPAQ in 1 M KCl at pH 9; the permeate side is 1 M aqueous KCl at pH 9. Photographs showing the colour change of feed and permeate solution at the beginning and finishing time in dialysis diffusion H-cells assembled with **d&g**, *s*PIM-SBF-1.40, **e&h,** *s*PIM-SBF-1.67, and **f&i,** Nafion 115 membrane.

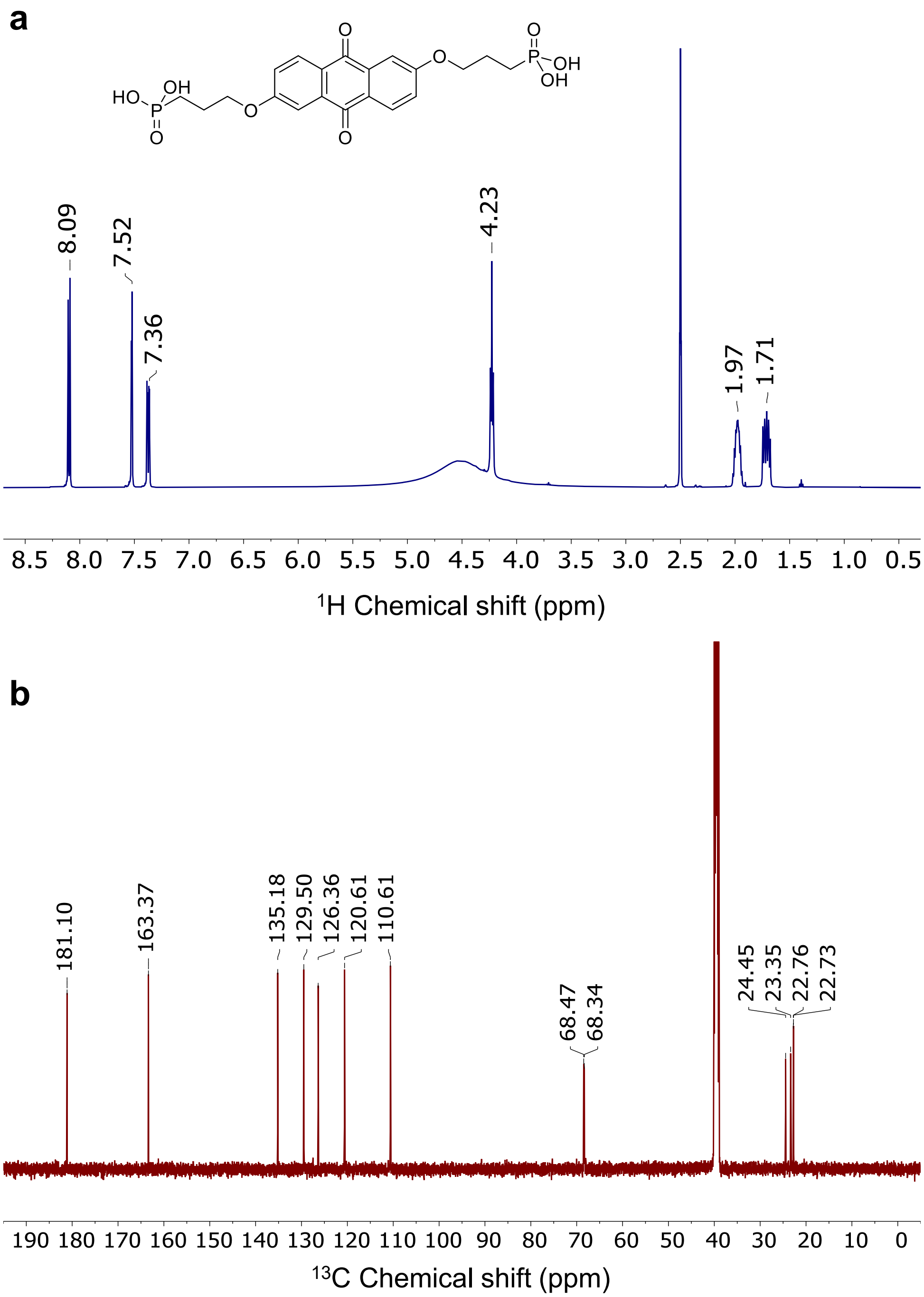

Supplementary Figure 28 | a, ^{1}H NMR and b, ^{13}C NMR spectra of 2,6-DPPAQ redox active molecules.

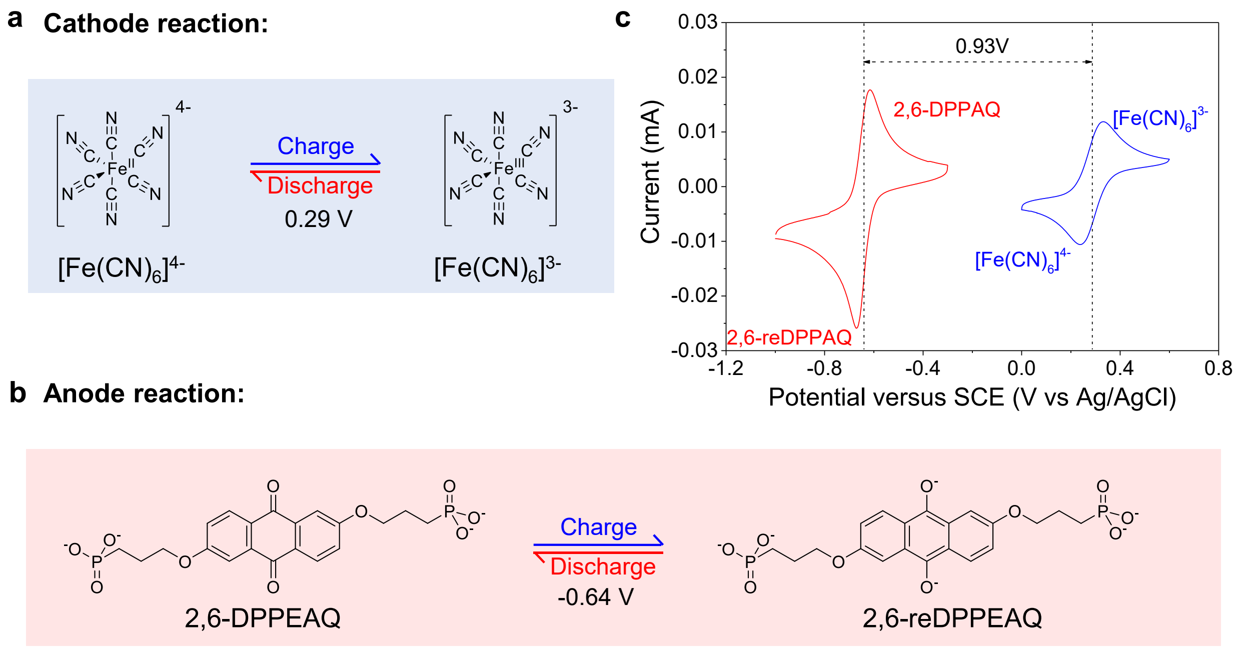

Supplementary Figure 29 | Electrochemical reaction mechanism of redox-active molecules. **a,** Cathode reaction based on $K_4Fe(CN)_6$|$K_3Fe(CN)_6$. **b,** Anode reaction based on2,6-DPPAQ|2,6-reDPPAQ. **c,** Cyclic voltammograms of 2,6-DPPAQ (red trace) and $K_4Fe(CN)_6$ (blue trace).

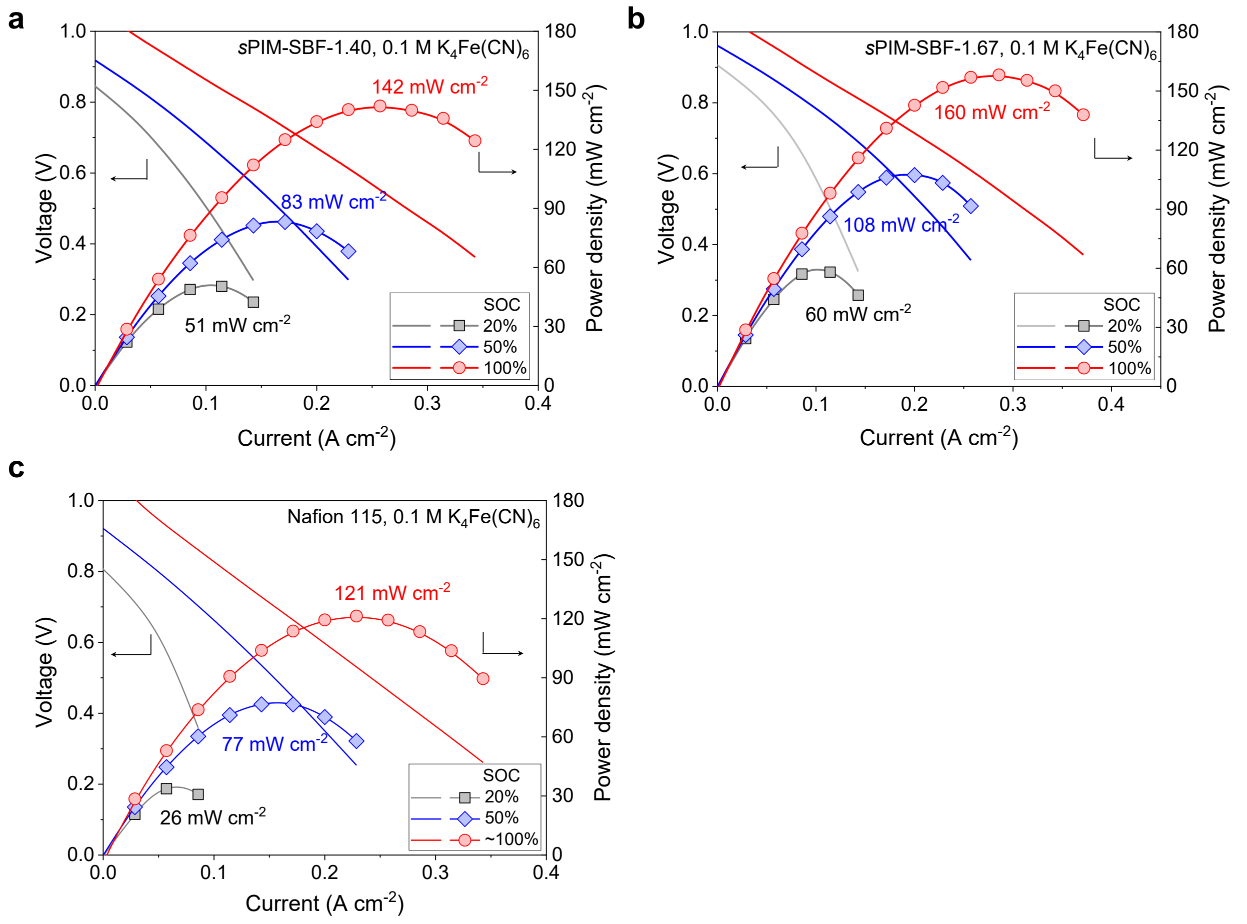

Supplementary Figure 30 | Polarization curves of RFB full cells at 20, 50, and ~100% SOC using 0.1 M $K_4Fe(CN)_6$| 0.1 M 2,6-DPPAQ redox couple and assembled with a, *s*PIM-SBF-1.40; b, *s*PIM-SBF-1.67 and c, Nafion-115 membranes. The thickness of measured membranes is around 150 µm. The temperature for RFB full cells measurements is around 30 °C.

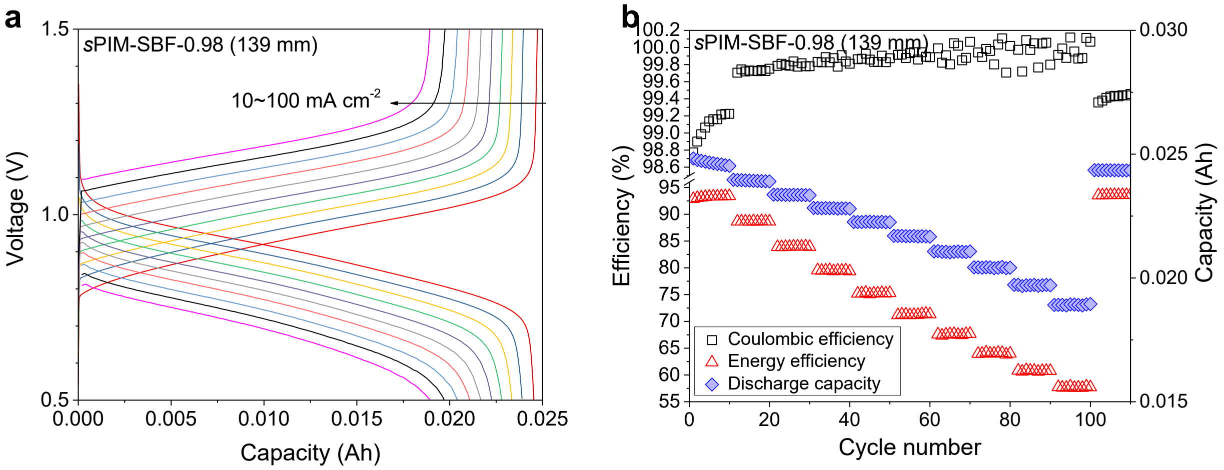

Supplementary Figure 31 | Low-concentration RFB full cell performance using 0.1 M $K_4Fe(CN)_6$ | 0.1 M 2,6-DPPAQ and assembled with *s*PIM-SBF-0.98 membrane (139 µm). a, Typical charging-discharging curves and **b,** discharge capacity, coulombic efficiency and energy efficiency versus cycling numbers of the RFB at current densities varied from 10 to 100 mA cm^{-2} with an increasing step of 10 mA cm^{-2} for each set of 10 cycles. The operating temperature is around 30 °C.

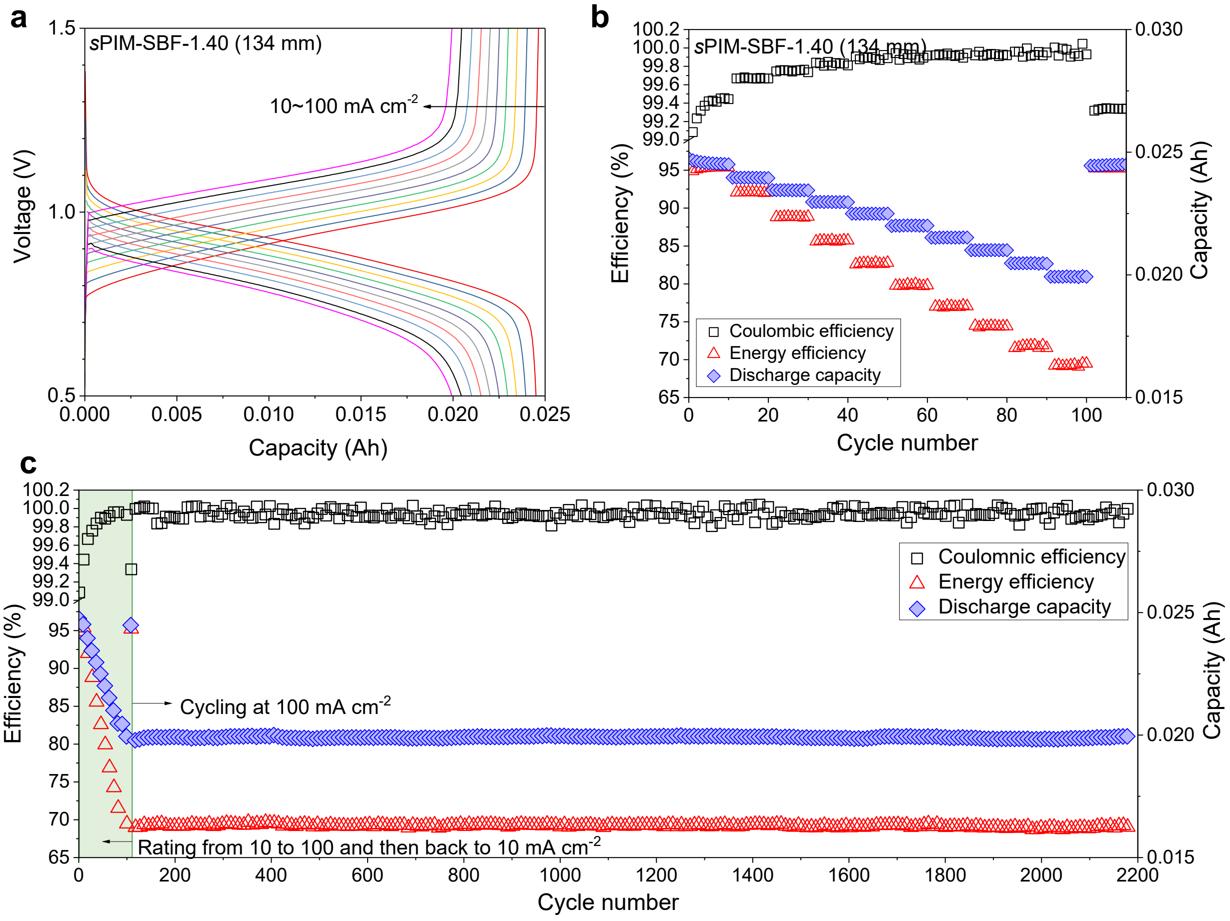

Supplementary Figure 32 | Low-concentration RFB full cell performance using 0.1 M $K_4Fe(CN)_6$ | 0.1 M 2,6-DPPAQ and assembled with *s*PIM-SBF-1.40 membrane (134 µm). a, Typical charging-discharging curves and **b,** discharge capacity, coulombic efficiency and energy efficiency versus cycling numbers of the RFB at current densities varied from 10 to 100 mA cm^{-2} with an increasing step of 10 mA cm^{-2} for each set of 10 cycles. **c,** Extended cycling performance for over 2100 cycles at 100 mA cm^{-2}. In Supplementary Figure 31c, the RFB are cycled at current density ranging from 10 to 100 mA cm^{-2} in the first 110 cycles (shaded in green colour) before cycling at a constant current density of 100 mA cm^{-2}. Cycling data is presented for every 20 cycles. The operating temperature is around 30 °C.

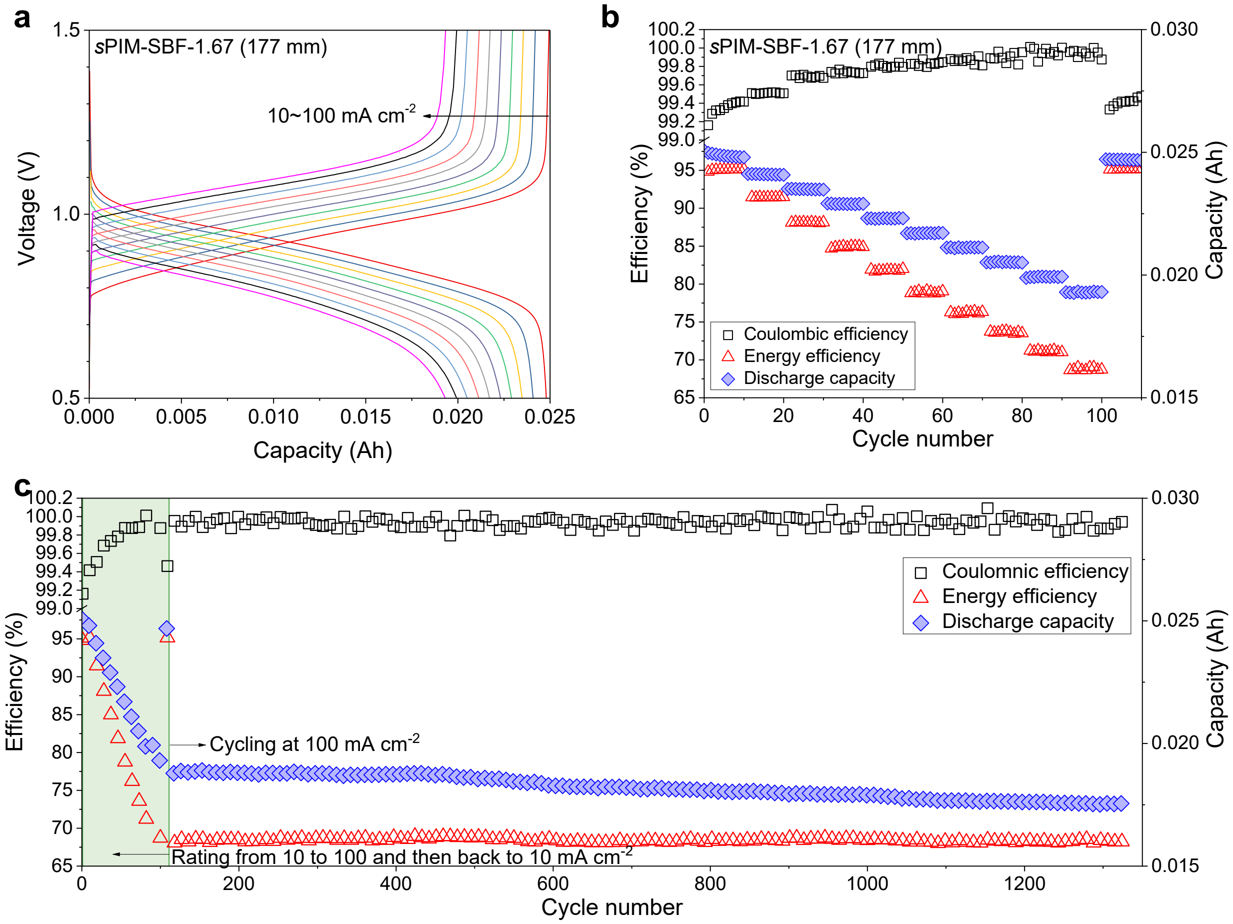

Supplementary Figure 33 | Low-concentration RFB full cell performance using 0.1 M $K_4Fe(CN)_6$ | 0.1 M 2,6-DPPAQ and assembled with *s*PIM-SBF-1.67 membrane (177 µm). a, Typical charging-discharging curves and **b,** discharge capacity, coulombic efficiency and energy efficiency versus cycling numbers of the RFB at current densities varied from 10 to 100 mA cm^{-2} with an increasing step of 10 mA cm^{-2} for each set of 10 cycles. **c,** Extended cycling performance for over 1300 cycles at 100 mA cm^{-2}. In Supplementary Figure 32c, the RFB are cycled at current density ranging from 10 to 100 mA cm^{-2} in the first 110 cycles (shaded in green colour) before cycling at a constant current density of 100 mA cm^{-2}. Cycling data is presented for every 20 cycles. The operating temperature was around 30 °C.

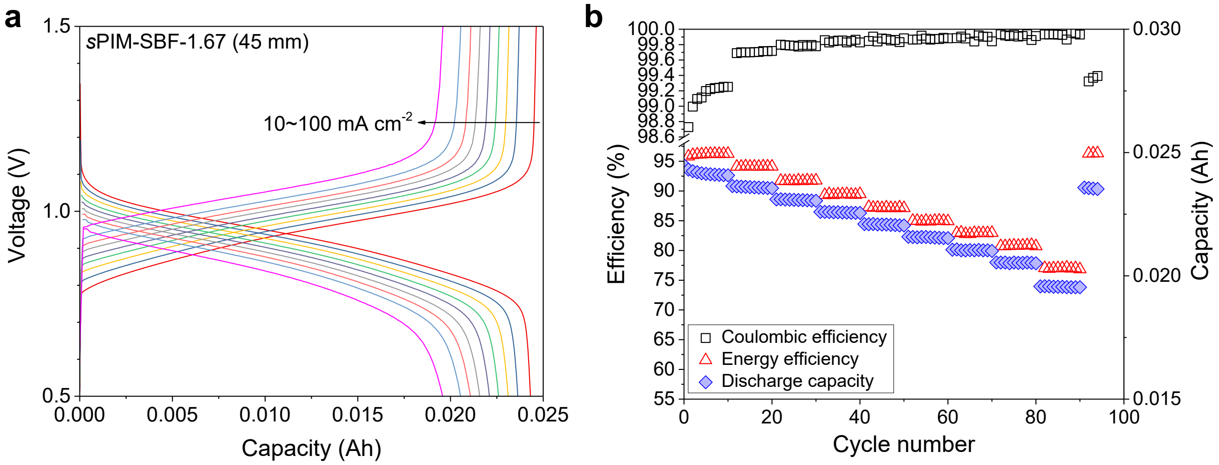

Supplementary Figure 34 | Low-concentration RFB full cell performance using 0.1 M $K_4Fe(CN)_6$ | 0.1 M 2,6-DPPAQ and assembled with thin free-standing *s*PIM-SBF-1.67 membrane (45 µm). a, Typical charging-discharging curves and **b,** discharge capacity, coulombic efficiency and energy efficiency versus cycling numbers of the RFB at current densities varied from 10 to 100 mA cm^{-2} with an increasing step of 10 mA cm^{-2} for each set of 10 cycles. The operating temperature was around 30 °C.

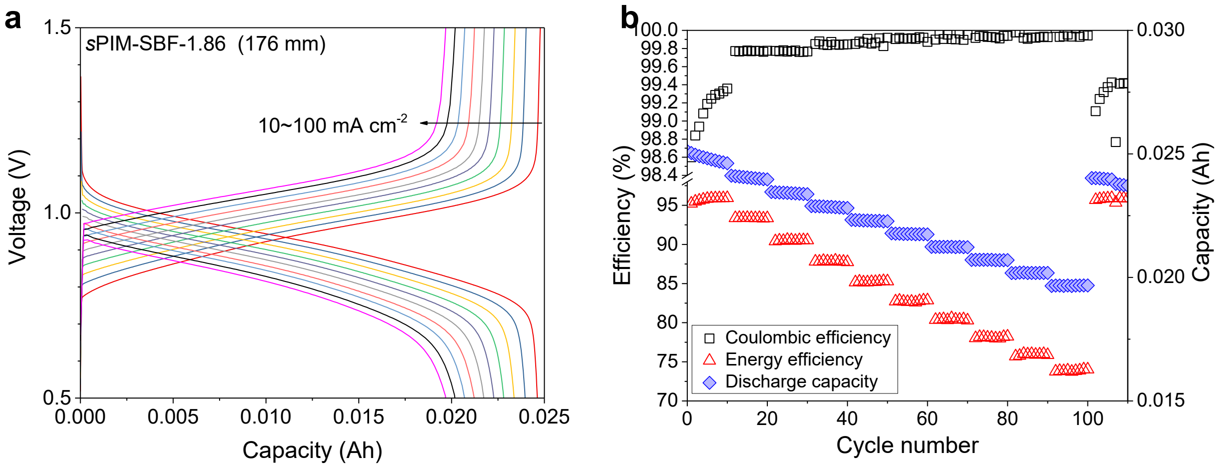

Supplementary Figure 35 | Low-concentration RFB full cell performance using 0.1 M $K_4Fe(CN)_6$ | 0.1 M 2,6-DPPAQ and assembled with thin free-standing *s*PIM-SBF-1.86 membrane (176 µm). a, Typical charging-discharging curves and **b,** discharge capacity, coulombic efficiency and energy efficiency versus cycling numbers of the RFB at current densities varied from 10 to 100 mA cm^{-2} with an increasing step of 10 mA cm^{-2} for each set of 10 cycles. The operating temperature was around 30 °C.

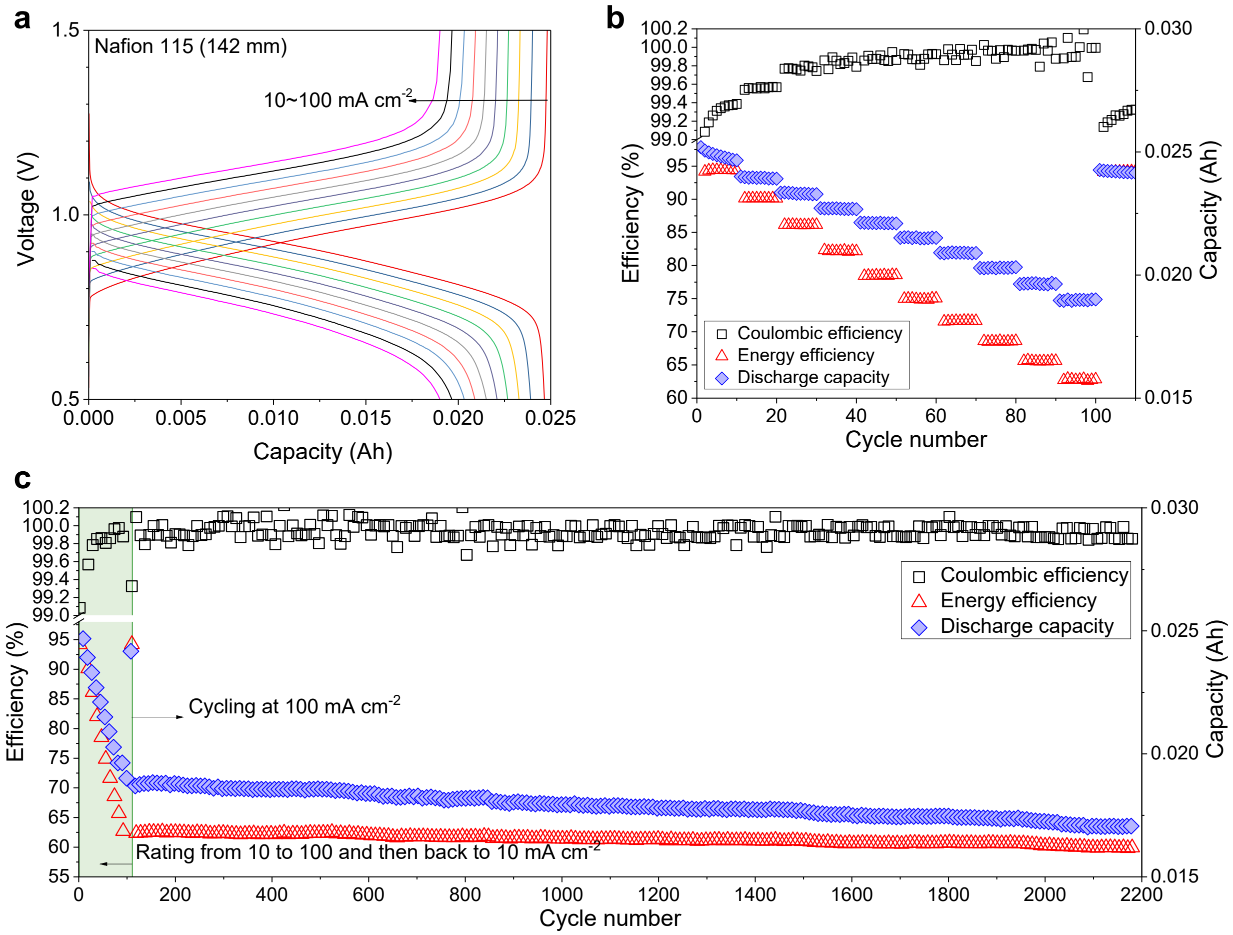

Supplementary Figure 36 | Low-concentration RFB full cell performance using 0.1 M $K_4Fe(CN)_6$ | 0.1 M 2,6-DPPAQ and assembled with Nafion 115 membrane (142 µm). a, Typical charging-discharging curves and **b,** discharge capacity, coulombic efficiency and energy efficiency versus cycling numbers of the RFB at current densities varied from 10 to 100 mA cm^{-2} with an increasing step of 10 mA cm^{-2} for each set of 10 cycles. **c,** Extended cycling performance for over 2100 cycles at 100 mA cm^{-2}. In Supplementary Figure 35c, the RFB are cycled at current density ranging from 10 to 100 mA cm^{-2} in the first 110 cycles (shaded in green colour) before cycling at a constant current density of 100 mA cm^{-2}. Cycling data is presented for every 20 cycles. The operating temperature was around 30 °C.

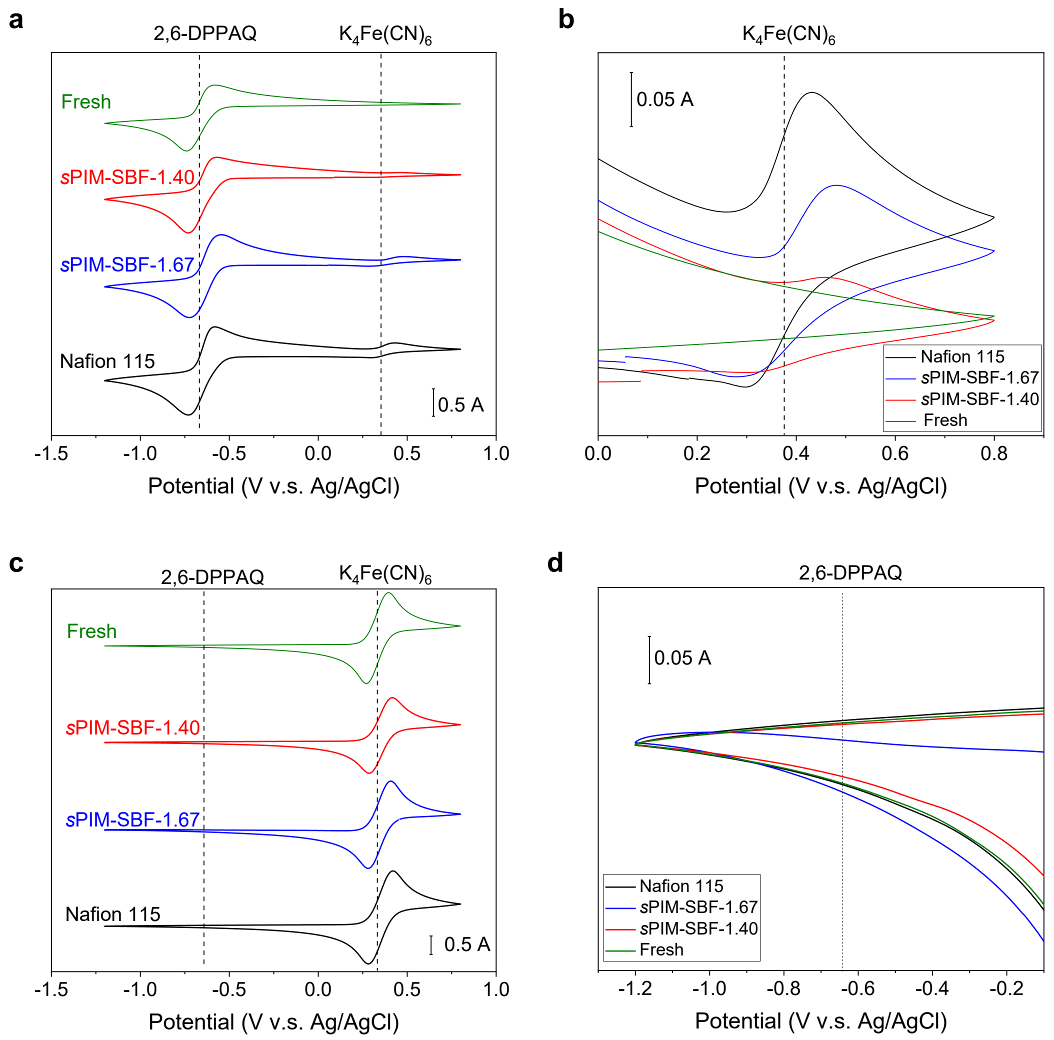

Supplementary Figure 37 | CV profiles of redox active molecules before and after RFB full cell cycling tests. a, CV profiles of 2,6-DPPAQ anolyte before and after battery cycling. **b,** Enlarged views from **a** to show permeated $K_4Fe(CN)_6$ redox peaks. **c,** CV profiles of $K_4Fe(CN)_6$ catholyte before and after battery cycling. **d,** Enlarged views from **c** showing no obvious 2,6-DPPAQ permeation. The testing temperature is around 25 °C.

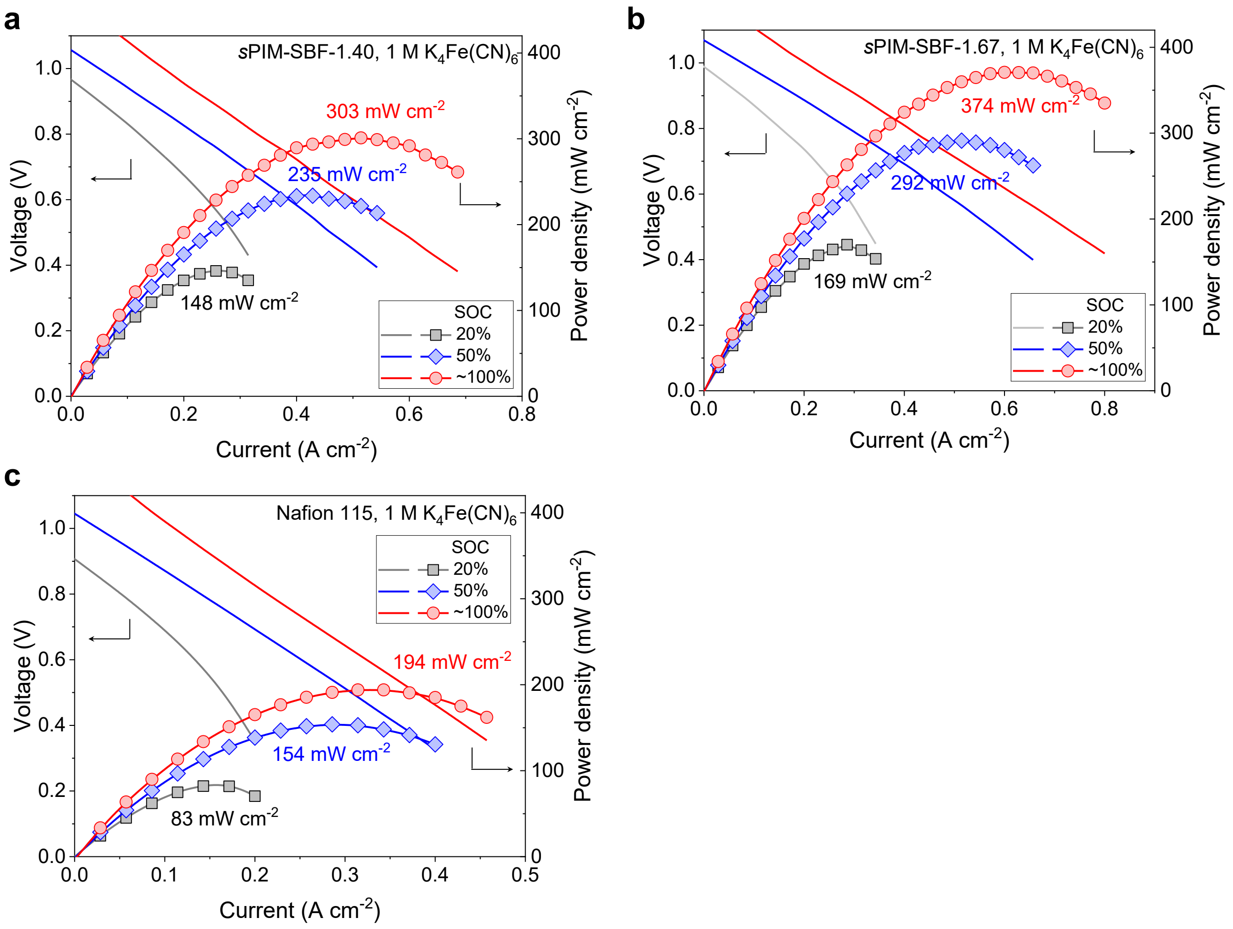

Supplementary Figure 38 | Polarization curves of RFB full cells at 20, 50, and ~100% SOC using 1 M $K_4Fe(CN)_6$| 0.4 M 2,6-DPPAQ redox couple and assembled with a, *s*PIM-SBF-1.40; b, *s*PIM-SBF-1.67 and c, Nafion-115 membranes. The thickness of measured membranes is around 150 µm. The temperature for RFB full cells measurements is around 30 °C.

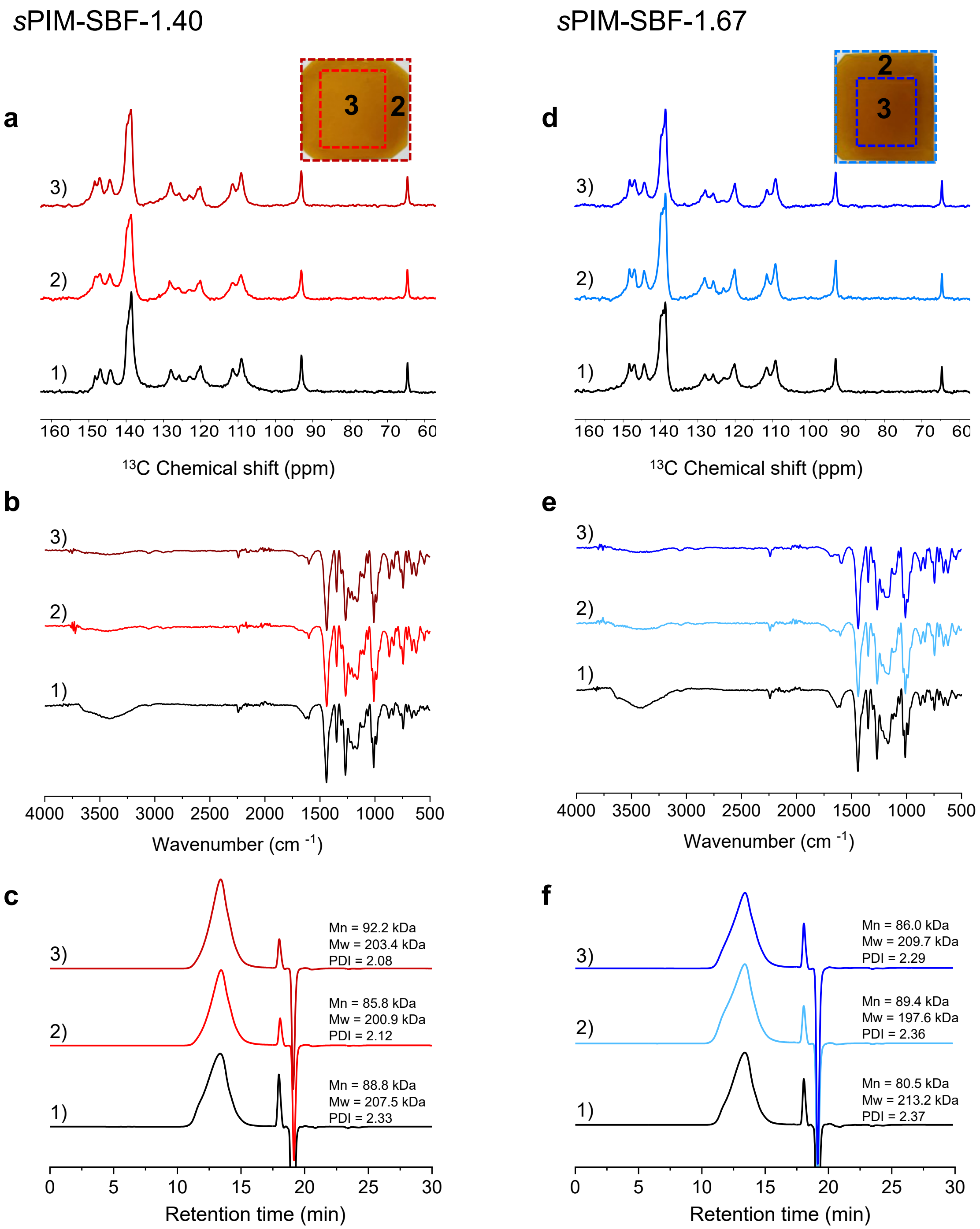

Supplementary Figure 39 | Chemical stability of *s*PIM-SBF membranes after RFB full cell cycling tests. a&d, liquid ^{13}C NMR, **b&e,** FT-IR and **c&f,** GPC of *s*PIM-SBF-1.40 membrane *s*PIM-SBF-1.67 membrane after being disassembled from RFB full cell cycling tests for around 46 months. 1) is the original polymer, 2) is the edge of used membranes without contacting with RFB electrolytes and 3) is the effective area of used membranes working in RFB cells, respectively. No structural change is observed in NMR and FT-IR spectra, and no degradation of polymer chains is observed according to GPC traces, indicating the good chemical stability of *s*PIM-SBF after long-term RFB cycling tests.

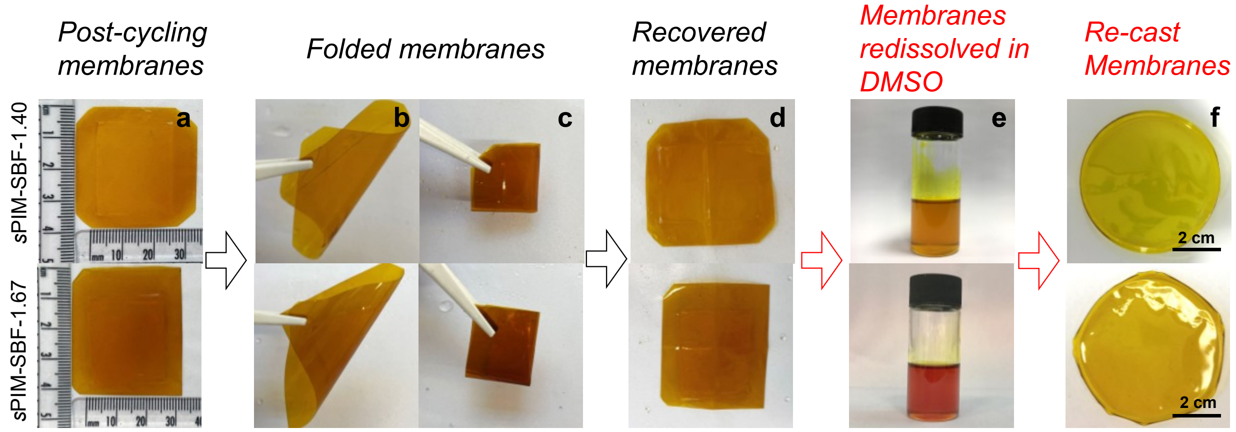

Supplementary Figure 40 | Mechanical stability and solution-processability of *s*PIM-SBF membranes after RFB full cell cycling tests. a-d, Photographs of *s*PIM-SBF-1.40 and *s*PIM-SBF-1.67 membrane after being disassembled from RFB full cell cycling tests for around 20 months. The post-cycling *s*PIM-SBF membranes maintain good integrity after being bent or fold repeatedly, showing excellent mechanical robustness. **e**, *s*PIM-SBF-1.40 and *s*PIM-SBF-1.67 solution by redissolving used membranes in DMSO. **f**, *s*PIM-SBF-1.40 and *s*PIM-SBF-1.67 membranes fabricated using recycled membrane solutions. The repeatable solution-processibility of used *s*PIM-SBF membranes after battery tests confirm their recyclability.

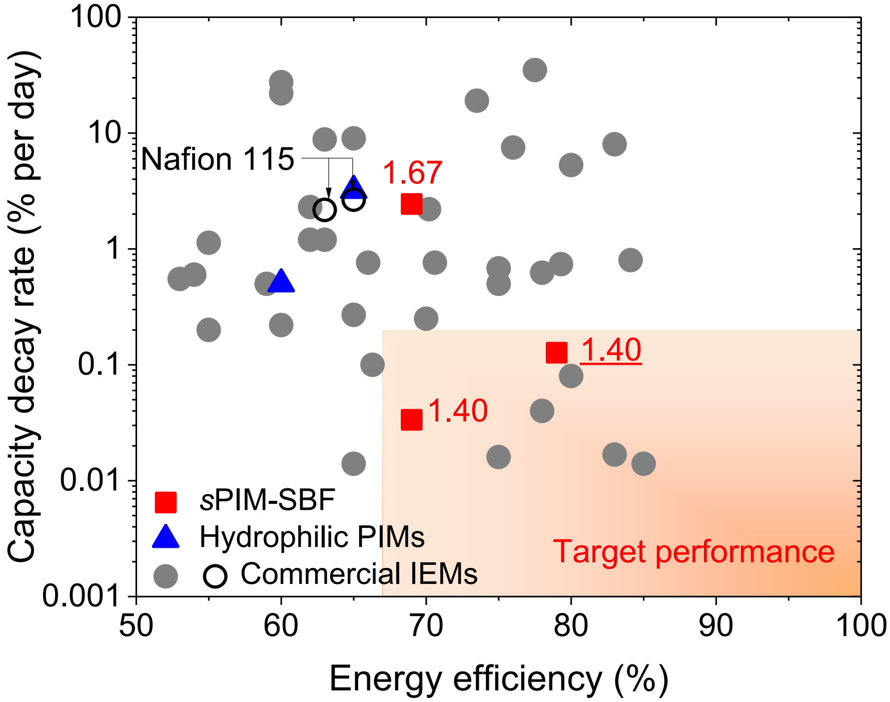

Supplementary Figure 41 | Energy efficiency versus capacity decay rate for *s*PIM-SBF membranes. Typical data reported in latest literature are included for comparisons such as those of commercial ion-exchange membranes (IEMs) including Nafion 212 and 115, Selemion DSV, AMV, AME 115, and CSO, and Fumasep E-610 (K) and FAP-375-PP. The orange area represents the target performance for an optimal aqueous organic RFB system. 1.40 marked in underline text represents the high-concentration RFB performance assembled with *s*PIM-SBF-1.40, while those of 1.40 and 1.67 left as plain text represent low-concentration RFB performance assembled with *s*PIM-SBF-1.40 and *s*PIM-SBF-1.67 membranes, respectively. (Extended data in Supplementary Table 14).

Supplementary Table 1 | Molecular weights (kDa) and PDI values of *s*PIM-SBF polymers.

Membranes	Mn	Mw	Mz	Mp	PDI
PIM-SBF	66.5	170.0	419.2	112.2	2.56
*s*PIM-SBF-0.53	80.0	178.0	390.0	112.4	2.25
*s*PIM-SBF-0.98	82.0	190.2	360.0	123.2	2.32
*s*PIM-SBF-1.40	88.8	207.5	412.1	116.4	2.33
*s*PIM-SBF-1.67	80.5	213.2	443.9	123.2	2.37
*s*PIM-SBF-1.86	89.7	216.6	403.5	132.7	2.41

Supplementary Table 2 | TGA results of *s*PIM-SBF polymers.

Polymers	Desulfonation step (T_{d1}[a], °C)	Backbone degradation (T_{d2}[b], °C)
PIM-SBF	-	612
*s*PIM-SBF-0.53	279	576
*s*PIM-SBF-0.98	295	562
*s*PIM-SBF-1.40	316	547
*s*PIM-SBF-1.67	345	544
*s*PIM-SBF-1.86	356	534

[a] T_{d1} is the peak degradation temperature for desulfonation step.
[b] T_{d2} is the peak degradation temperature for backbone degradation.

Supplementary Table 3 | Physical properties of PIM-SBF and *s*PIM-SBF polymers.

Polymers	IEC (mmol g^{-1})	SO_3K per SBF unit (/)	SA_{BET}[a] (m^2 g^{-1})	Micropore volume[b] (cm^3 g^{-1})	Skeletal density (g cm^{-3})	Envelop density (g cm^{-3})	Electrolyte uptake (wt.%)	Linear swelling ratio (%)
PIM-SBF	-	-	736	0.227	1.3269 ± 0.0044	1.06	10.9 ± 0.4	0.6 ± 0.02
*s*PIM-SBF-0.53	0.53±0.01	0.28±0.01	692	0.217	1.3515 ± 0.0029	0.791	26.9 ± 2.2	6.5 ± 0.3
*s*PIM-SBF-0.98	0.98±0.09	0.56±0.06	634	0.203	1.3786 ± 0.0025	0.991	44.6 ± 1.8	9.0 ± 0.5
*s*PIM-SBF-1.40	1.40±0.07	0.84±0.05	538	0.182	1.4261 ± 0.0016	0.994	61.5 ± 2.6	15.6 ± 1.1
*s*PIM-SBF-1.67	1.67±0.16	1.04±0.12	490	0.146	1.4416 ± 0.0026	1.09	96.8 ± 3.9	24.1 ± 0.9
*s*PIM-SBF-1.86	1.86±0.02	1.20±0.02	482	0.167	1.4802 ± 0.0022	1.23	101 ± 4.0	32.0 ± 1.8

[a]SA_{BET} was obtained by nitrogen adsorption in the range of P/P_0 = 0.001-0.1.
[b]Micropore volume was obtained from nitrogen adsorption based on the NLDFT model.

Supplementary Table 4 | Polymerisation parameters and final results of the polymerisation algorithm. Results are averaged over five independent systems, with standard deviations shown in brackets.

Polymers	IEC (g mmol^{-1})	SO_3H per monomer	Initial Box Length (Å)	Initial Packing Density (g cm^{-3})	Degree of polymerisation (%)	Final Bulk Density (g cm^{-3})
PIM-SBF	0	0	70.0	0.366	96 (0.02)	0.96 (0.03)
*s*PIM-SBF-0.53	0.53	0.28	71.0	0.365	95 (0.01)	1.04 (0.02)
*s*PIM-SBF-0.98	0.98	0.56	72.0	0.365	95 (0.02)	1.09 (0.02)
*s*PIM-SBF-1.40	1.40	0.84	73.0	0.365	94 (0.01)	1.11 (0.02)
*s*PIM-SBF-1.67	1.67	1.04	73.5	0.367	94 (0.01)	1.12 (0.01)
*s*PIM-SBF-1.86	1.86	1.20	74.0	0.367	95 (0.02)	1.14 (0.02)

Supplementary Table 5 | Results of the simulated pore volume with respect to a helium sized atom (radius = 1.3 Å), and the simulated and experimental skeletal densities. Standard deviations are reported in brackets.

Polymers	v_{pore} (cm^3 g^{-1})	ρ_{skel} Sim (g cm^{-3})	ρ_{skel} Exp (g cm^{-3})
PIM-SBF	0.12 (0.02)	1.09 (0.01)	1.33
*s*PIM-SBF-0.53	0.08 (0.01)	1.14 (0.00)	1.35
*s*PIM-SBF-0.98	0.07 (0.01)	1.16 (0.01)	1.38
*s*PIM-SBF-1.40	0.06 (0.01)	1.19 (0.01)	1.43
*s*PIM-SBF-1.67	0.06 (0.01)	1.20 (0.00)	1.44
*s*PIM-SBF-1.86	0.05 (0.01)	1.21 (0.01)	1.48

Supplementary Table 6 | Largest cavity diameter (D_i) and largest free sphere paths (D_f) of the PIM-SBF and *s*PIM-SBF systems. Standard deviations across the 5 independent models are reported in brackets.

Polymers	Largest Cavity Diameter (D_i) (Å)	Largest Free Sphere Path (D_f) (Å)
PIM-SBF	10.7 (1.0)	4.0 (0.3)
*s*PIM-SBF-0.53	9.7 (0.6)	3.6 (0.4)
*s*PIM-SBF-0.98	9.3 (1.0)	3.4 (0.2)
*s*PIM-SBF-1.40	9.1 (0.9)	3.3 (0.4)
*s*PIM-SBF-1.67	9.5 (0.9)	3.1 (0.2)
*s*PIM-SBF-1.86	8.7 (0.7)	3.2 (0.3)

Supplementary Table 7 | Mechanical property results of *s*PIM-SBF polymers.

Polymers	Young's Modulus (MPa)	Tensile strength (MPa)	Elongation at break (%)
PIM-SBF	1176	70.9	37.8
*s*PIM-SBF-0.98	887	45.3	44.8
*s*PIM-SBF-1.40	867	46.4	40.5
*s*PIM-SBF-1.67	545	39.1	75.6
*s*PIM-SBF-1.86	558	29.8	73.4

Supplementary Table 8 | Membrane resistance (R_m, Ω cm^2) and corresponding ionic conductivity (σ, 10^{-3} S cm^{-1}) of *s*PIM-SBF and Nafion membranes.

Membranes	R_m in KCl [a]	Apparent σ [a]	R_m in DI water [b]	Intrinsic σ [b]
*s*PIM-SBF-0.53	2.882	2.7	5.699	1.5
*s*PIM-SBF-0.98	0.698	11.3	1.256	5.5
*s*PIM-SBF-1.40	0.429	19.5	1.029	9.0
*s*PIM-SBF-1.67	0.274	31.2	0.593	13.7
*s*PIM-SBF-1.86	0.244	39.4	0.500	16.9
Nafion 115	0.628	11.9	2.348	3.2

[a] Membrane resistance and derived apparent ionic conductivity measured in 1 M aqueous KCl at 30 °C by EIS.
[b] Membrane resistance and derived intrinsic ionic conductivity measured in deionised water at 30 °C by EIS.

Supplementary Table 9 | Ion permeance rate[a] (J_x, mol m^{-2} h^{-1}), permeability (P_x, 10^{-10} cm^2 s^{-1}) and their corresponding selectivities with respect to $K_4Fe(CN)_6$ (P_x/ $P_{K_4Fe(CN)_6}$) for *s*PIM-SBF and Nafion 115 membranes.

Membranes	J_{KCl}	J_{KNO_3}	$J_{K_2SO_4}$	$J_{K_2CO_3}$
*s*PIM-SBF-0.53	0.054 ± 0.016	0.04 ± 0.007	0.000097	0.000053
*s*PIM-SBF-0.98	0.12 ± 0.04	0.12 ± 0.02	0.0007	0.00039
*s*PIM-SBF-1.40	0.40 ± 0.08	0.33 ±0.09	0.0055 ± 0.0009	0.0021 ±0.0005
*s*PIM-SBF-1.67	0.93 ± 0.16	0.94 ±0.14	0.019 ±0.003	0.011 ±0.002
*s*PIM-SBF-1.86	1.8 ± 0.15	1.8 ±0.31	0.059 ±0.010	0.038 ± 0.004
Nafion 115	0.14 ± 0.02	0.2 ± 0.05	0.025 ±0.007	0.017 ± 0.003
Membranes	P_{KCl} ($P_{KCl}/P_{K_4Fe(CN)_6}$)	P_{KNO_3} ($P_{KNO_3}/P_{K_4Fe(CN)_6}$)	$P_{K_2SO_4}$ ($P_{K_2SO_4}/P_{K_4Fe(CN)_6}$)	$P_{K_2CO_3}$ ($P_{K_2CO_3}/P_{K_4Fe(CN)_6}$)
*s*PIM-SBF-0.53	2200 (240,000)	1600 (180,000)	4 (440)	2.2 (240)
*s*PIM-SBF-0.98	5600 (19,000)	5400 (19,000)	32 (110)	17 (61)
*s*PIM-SBF-1.40	16000 (9,400)	13000 (7,800)	220 (130)	82 (48)
*s*PIM-SBF-1.67	44000 (1,700)	45000 (1,700)	890 (34)	510 (19)
*s*PIM-SBF-1.86	74000 (1,100)	76000 (1,100)	2500 (37)	1600 (24)
Nafion 115	5400 (260)	7900 (380)	1000 (48)	680 (33)

[a] 0.4 M KCl, 0.4 M KNO_3, 0.2 M K_2SO_4, or 0.2 M K_2CO_3 as a feed solution and deionised H_2O as a permeate solution.

Supplementary Table 10 | Permeance rate (mol m^{-2} h^{-1}) and permeability (cm^{2} s^{-1}) of redox-active molecules through *s*PIM-SBF and Nafion membranes.

Membranes	$K_4Fe(CN)_6$[a]		2,6-DPPAQ[a]		2,6-DHAQ[b]	
	Permeance rate	Permeability	Permeance rate	Permeability	Permeance rate	Permeability
*s*PIM-SBF-0.53	2.2×10^{-7}	1.1×10^{-12}	-	-	-	-
*s*PIM-SBF-0.98	6.4×10^{-6}	2.6×10^{-11}	-	-	7.1×10^{-5}[c]	2.4×10^{-10}
*s*PIM-SBF-1.40	4.3×10^{-5}	1.7×10^{-10}	1.7×10^{-7}	6.3×10^{-13}	8.4×10^{-4}[c]	9.1×10^{-10}
*s*PIM-SBF-1.67	5.5×10^{-4}	2.3×10^{-9}	3.0×10^{-7}	1.3×10^{-12}	1.1×10^{-3}[c]	6.0×10^{-9}
*s*PIM-SBF-1.86	1.6×10^{-3}	7.5×10^{-9}	-	-	-	-
Nafion 115	5.1×10^{-4}	2.2×10^{-9}	4.6×10^{-7}	2.0×10^{-12}	2.3×10^{-5}	9.9×10^{-11}

[a] 0.1 M $K_4Fe(CN)_6$ or 0.1 M 2,6-DPPAQ in 1M KCl at pH 9 as a feed solution and 1 M aqueous KCl at pH 9 as a permeate solution.

[b] 0.1 M 2,6-DHAQ in 1M aqueous KOH as a feed solution and 1 M aqueous KOH as a permeate solution.

[c] The thickness of *s*PIM-SBF-0.98, *s*PIM-SBF-1.40, and *s*PIM-SBF-1.67 membranes is 119, 39 and 190 µm, respectively. Other permeance rate were evaluated from ~ 150-um-thick *s*PIM-SBF membranes.

Supplementary Table 11| Parameters for lifetime estimation.

Parameters	*s*PIM-SBF-1.40	Nafion 115
Total active area of cell stack, *A*	1 m^2	
Open circuit voltage	1 V	
Targeted energy storage capacity of cell stack (equivalent number of electrons)	2 kWh (74.6 mol)	
Charging-discharging duration time in cell stack	4 h	
Calendar time amplification factor, *a*	6 (24h / 4h)	
Concentration of redox species in cell stack	1 M	
Current density	0.1 A cm^{-2}	
Operation time	One cycle per day	
Capacity loss at scrap	20%	
Utilization of actives	93%	88%
Required number of electrons, *C*	80.2 mol	84.8 mol
Catholyte volume	80.2 L	84.8 L
Capacity decay rate in lab cells, *P*	3.78×10^{-4} mol m^{-2} h^{-1}	7.92×10^{-3} mol m^{-2} h^{-1}
Estimated lifetime	***29.1*** calendar years	***1.47*** calendar years

Supplementary Table 12 | Cost analysis of *s*PIM-SBF polymer production. The yield of intermediates/products are reported in brackets.

Raw Materials	Price in £ & vendors	Intermediates/products
Veratrole<br>(1 equiv., 50.0 g)	£ 0.05/g<br>Alfa Aesar	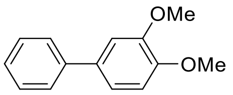 **Compound 1** (75 %)
Bromine<br>(1 equiv., 57.8 g)	£ 0.08/g<br>Alfa Aesar	
Benzene boronic acid<br>(1 equiv., 44.1 g)	£ 0.19/g<br>Acros Organics	
$Pd(PPh_3)_4$<br>(0.3 % mol, 1.2 g)	£ 7.4/g<br>Fluorochem	
Compound 1<br>(1 equiv., 58.1 g)		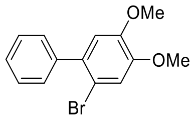 **Compound 2** (90 %)
Bromine<br>(1 equiv., 43.3 g)	£ 0.08/g<br>Alfa Aesar	
Compound 2<br>(1 equiv., 40.5 g/71.2 g in total)		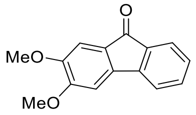 **Compound 3** (76 %)
n-butyl lithium 2.5 M in hexane<br>(1.05 equiv., 58.2 mL)	£ 0.08/mL<br>Acros Organics	
Methanesulfonic acid<br>(22 w/v%, 180 mL)	£ 0.09/mL<br>Acros Organics	
Compound 2<br>(1 equiv., 30.7 g)		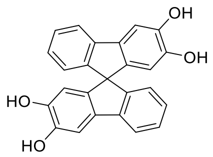 **Compound 4** (70 %)
Compound 3<br>(1 equiv., 25.3 g)		
n-butyl lithium 2.5 M in hexane<br>(1.05 equiv., 44.2 mL)	£ 0.08/mL<br>Acros Organics	
Boron tribromide<br>(3 equiv., 55.4 g)	£ 0.40/g<br>Merck Life Sciences	
Compound 4<br>(1 equiv., 28.0 g)		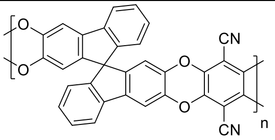 **PIM-SBF** (90 %)
2,3,5,6-tetrafluoroterephthalonitrile<br>(1 equiv., 14.7 g)	£ 0.14/g<br>Fluorochem	
PIM-SBF<br>(1 equiv., 33.2 g)		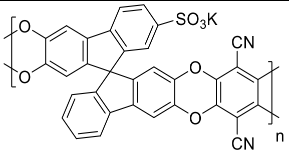 ***s*PIM-SBF-1.40** (98 %)
Trimethylsilyl chlorosulfonate<br>(1.7 equiv., 17.4 mL)	£ 1.1/mL<br>Merck Life Sciences	
Total cost: £ 95.6	Yield: 40.2 g	**Price: £ 2.4/g**

The production cost of *s*PIM-SBF-1.40 was estimated based on the cost of raw materials, reagents and catalysts used in the synthesis (cost data were accessed on websites of common chemical vendors on 01 Dec. 2021). The final cost would be £ 2.4 per gram for sPIM-SBF polymers and £ 120 per m^2 for *s*PIM-SBF membranes (50 μm thick). It should be noted that this simple estimation does not include the costs associated with manufacturing and operational costs. We envision that large scale synthesis and manufacturing will further reduce the cost of these polymer membranes.

Supplementary Table 13 | Key ion/redox-active molecule transport properties and RFB parameters of representative *s*PIM-SBF and Nafion 115 membranes.

Membranes	KCl conductivity (10^{-3} S cm^{-1})[a]	Permeation rate (mol m^{-2} h^{-1})			Selectivity ($\boldsymbol{P}_{KCl}/\boldsymbol{P}_{K4Fe(CN)6}$)	R_m (Ω cm^2)[b]	Energy efficiency(%)	Power density (mW cm^{-2})	Capacity decay per cycle % (per day %)
		KCl	$K_4Fe(CN)_6$	2,6-DPPAQ					
*s*PIM-SBF-1.40	20	0.40	4.3×10^{-5}	1.7×10^{-7}	9400	1.2	69	142[c]	0.0000795 (0.0335)[c]
							79	303[d]	0.00189 (0.127)[d]
*s*PIM-SBF-1.67	31	0.93	5.5×10^{-4}	3.0×10^{-7}	1700	1.1	69	160[c]	0.00472 (2.17)[c]
							N/A	374[d]	N/A
Nafion 115	12	0.14	5.1×10^{-4}	4.6×10^{-7}	260	1.6	63	121[c]	0.00535 (2.44)[c]
							65	194[d]	0.0355 (2.66)[d]

[a] Ionic conductivity was measured in 1 M KCl aqueous solution at pH = 9 and 30 °C.
[b]Membrane resistance (R_m) obtained from 0.1 M $K_4Fe(CN)_6$ | 0.1 M 2,6-DPPAQ RFB full cells by high-frequency EIS at around 30 °C.
[c]Low-concentration RFB full cell performance using 0.1 M $K_4Fe(CN)_6$ and 0.1 M 2,6-DPPAQ at around 30 °C.
[d]High-concentration RFB full cell performance using 1 M $K_4Fe(CN)_6$ and 0.4 M 2,6-DPPAQ at around 30 °C.

Supplementary Table 14 | Summary of organic aqueous RFB systems.

Anolytes | Catholytes	Membrane	Electron conc. (M)	Current density (mA cm^{-2})	Energy efficiency (%)	Capacity decay rate		pH	Author/year
					% per cycle	% per day		
$Zn(OH)_4^{2-}$ | FQH_2	Nafion 117 & Selemion DSV	0.2	20	70.2	0.08	2.2	-0.7	Park/2019[16]
V^{3+} | MB	Fumasep FAP-375-PP	3.0	80	66[b]	0.074	0.76	-0.5	Zhang/2019[17]
AQDS | HBr	Nafion 115	2	250	62[b]	0.1	1.2	0	Chen/2016[18]
AQS | HBr	Nafion 212	2	250	73.5	1	19	0	Gerhardt/2017[19]
PEGAQ | $K_4Fe(CN)_6$	Fumasep E-620(K)	3	50	75[b]	0.043	0.5	7	Jin and Jing/2019[20]
MV polymer | Tempo polymer	Dialysis membrane	0.37	40	76	0.25	7.5	7	Janoschka/2015[21]
MV | 4-OH-TEMPO	Selemion	0.5	60	60[b]	0.1	27.5	7	Liu/2016[22]
MV | TEMPTMA	Fumasep FAA-3-PE-3	2	80	65[b]	0.026	0.27	7	Janoschka/2016[23]
$(NPr)_2V$ | N^{Me}-TEMPO	Selemion AMV	0.5	60	55[b]	0.005	0.2	7	Hu/2018[24]
$[(NPr)_2TTz]Cl_4$ | N^{Me}-TEMPO	Selemion AMV	0.1	40	70	0.03	0.25	7	Luo/2018[25]
BTMAP-Vi | TMAP-TEMPO	Selemion AMV	0.1	40	78[b]	0.007	0.624	7	Liu/2019[26]
		1.5	100	53[b]	0.015	0.55	7	
BTMAP-Vi | 4-OH-TEMPO	Selemion AMV	0.1	40	80[b]	0.041	5.3	7	Liu/2019[26]
BTMAP-Vi | BTMAP-Fc	Selemion DSV	1.3	50	66.3	0.0057	0.1	7	Beh/2017[27]
$[(NPr)_2V]Br_4$ | FcNCl	Selemion AME 115	0.5	60	59	0.01	0.5	7	DeBruler/2017[28]
$[(Me)(NPr)V]Cl_3$ | FcNCl	Selemion AME 115	0.5	60	63	0.18	8.8	7	DeBruler/2017[28]
MV | FcNCl	Selemion AMV	0.5	60	60	0.01	0.22	7	Hu/2017[29]
Diquat | FcNCl	Selemion AMV	0.5	5	84.1	0.2	0.8	7	Huang/2018[30]
$(SPr)_2V$ | $(NH_4)_4Fe(CN)_6$	Selemion CSO	0.5	40	83	0.0003	0.0168	7	Luo/2019[31]
BHOP-Vi | FcNCl	Selemion DSV	2.0	100	55[b]	0.055	1.13	7	Liu/2020[32]
2,6-DPPEAQ | $K_4Fe(CN)_6$	Fumasep E-620(K)	1	100	65[b]	0.0004	0.014	9	Ji/2019[33]
BPP-Vi | $K_4Fe(CN)_6$	Fumasep E-620(K)	1	60	75[b]	0.00069	0.016	9	Jin/2020[34]
2,6-DBEAQ | $K_4Fe(CN)_6$	Fumasep E-620(K)	1	100	78[b]	0.001	0.04	12	Kwabi/2018[35]
FMN | $K_4Fe(CN)_6$	Nafion 212	0.48	80	54[b]	0.01	0.6	14	Orita/2016[36]
DHPS | $K_4Fe(CN)_6$	Nafion 212	2.8	100	>75	0.0195	0.68	14	Hollas/2018[37]

Anolytes | Catholytes	Membrane	Electron conc. (M)	Current density (mA cm^{-2})	Energy efficiency (%)	Capacity decay rate		pH	Author/year
					% per cycle	% per day		
ACA | $K_4Fe(CN)_6$	Nafion 212	1.0	100	63	0.013	1.2	14	Lin/2016[38]
2,6-DHAQ | $K_4Fe(CN)_6$	Nafion 212	1	100	83[b]	0.1	8	14	Lin/2015[39]
DHBQ | $K_4Fe(CN)_6$	Nafion 115	1	100	65	0.24	9	14	Yang and Tong/2018[40]
DMBQ | $K_4Fe(CN)_6$	Nafion 115	0.2	60	55-65	0.11	22	14	Sun/2019[41]
DMOBQ | $K_4Fe(CN)_6$	Nafion 115	0.2	60	75-80	0.17	35	14	Sun/2019
Bislawsone | $K_4Fe(CN)_6$	Fumasep E-620(K)	2	100	79.3	0.038	0.74	14	Tong/2019[42]
DPivOHAQ | $K_4Fe(CN)_6$	Fumasep E-620(K)	1	100	85[b]	0.00031 (-)	0.014 (0.0018)	12 (14)	Wu/2020[43]
BHPC | $K_4Fe(CN)_6$	Nafion 212	1.0	100	~80	0.014	0.08	14	Wang/2020[44]
4C7SFL | $K_4Fe(CN)_6$[c]	Nafion 212	2.0	100	70.6	0.02	0.76	14	Feng/2021[45]
**2,6-DPPAQ | $K_4Fe(CN)_6$**	**Nafion 115**	**0.1**	**100**	**63**	**0.00472**	**2.17**	**9**	**This work**
		**1.0**	**100**	**65**	**0.0355**	**2.66**	**9**	
	***s*PIM-SBF-1.86**	**0.1**	**100**	**74**	**-**	**-**	**9**	
	***s*PIM-SBF-1.67**	**0.1**	**100**	**69**	**0.00535**	**2.44**	**9**	
	***s*PIM-SBF-1.40**	**0.1**	**100**	**69**	**0.0000795**	**0.0335**	**9**	
		**1.0**	**100**	**79**	**0.00189**	**0.127**	**9**	
	***s*PIM-SBF-0.98**	**0.1**	**100**	**58**	**-**	**-**	**9**	

[a]Capacity decay rates from potentiostatic cycling or galvanostatic cycling with potentiostatic finish are marked in underline text, while these results from pure-galvanostatic cycling are left as plain text. [b]Estimated from figures in the literatures. [c]4C7SFL | $K_4Fe(CN)_6$ cell is operated at 50 °C, whereas other RFB full cells run at R.T. FQH_2 = 2,3,5,6-tetrakis((dimethylamino)methyl)hydroquinone; MB = methylene blue; AQDS = anthraquinone disulfonic acid; AQS = anthraquinone monosulfonic acid; PEGAQ = 1,8-bis(2-(2-(2-hydroxyethoxy)ethoxy)ethoxy)anthracene-9,10-dione; MV = methyl viologen; 4-HO-TEMPO = 4-hydroxy-2,2,6,6-tetramethylpiperidin-1-oxyl; N^{Me}-TEMPO (TEMPTMA) = 4-trimethylammonium-TEMPO; $(NPr)_2V$ = 1,10-bis[3-(trimethylammonio)-propyl]-4,40-bipyridinium tetrabromide; $[(NPr)_2TTz]Cl_4$ = 4,4′-(thiazolo[5,4-d]thiazole-2,5-diyl)bis(1-(3 (trimethylammonio)propyl)pyridin-1-ium) tetrachloride; BTMAP-Vi ($[(NPr)_2V]Cl_4$) = bis(3-trimethylammonio)-propyl viologen tetrachloride; TMAP-TEMPO = 4-[3-(trimethylammonio)propoxy]-2,2,6,6-tetramethylpiperidine-1-oxyl chloride; BTMAP-Fc = bis((3 trimethylammonio)propyl)ferrocene dichloride; FcNCl = (ferrocenylmethyl)trimethylammonium chloride; $[(NPr)_2V]Br_4$ = 1,10-Bis[3-(trimethylamonium)propyl]-4,40-bipyridinium Tetrabromide; $[(Me)(NPr)V]Cl_3$ = 1-methyl-10-[3-(trimethylammonio)propyl]-4,40-bipyridi-nium trichloride; $(SPr)_2V$ = 1,10-bis(3-sulfonatopropyl)-4,40-bipyridinium; BHOP-Vi=1,1'-bis(3-hydroxypropyl) viologen dibromide; 2,6-DPPEAQ (2,6-DPPAQ) = (((9,10-dioxo-9,10-dihydroanthracene-2,6-diyl) bis(oxy))bis(propane-3,1-diyl))bis(phosphonic acid); BPP-Vi = 1,1′-bis(3-phosphonopropyl)-[4,4′-bipyridine]-1,1′-diium dibromide; 2,6-DBEAQ = 4,4′-((9,10-anthraquinone-2,6-diyl)dioxy)dibutyrate; FMN = Flavin mononucleotide; DHPS = 7,8-dihydroxyphenazine-2-sulfonic acid; ACA = Alloxazine7/8-carboxylic acid; 2,6-DHAQ = 2,6-dihydroxanthraquinone; DHBQ = dihydroxybenzoquinone; DMBQ = 2,5-dihydroxy-3,6-dimethyl-1,4-benzoquinone; DMOBQ = 2,5-dimethoxy-3,6-dihydroxy-1,4-benzoquinone; DPivOHAQ= 3,30-(9,10-Anthraquinone-Diyl)Bis(3-Methylbutanoic Acid); BHPC = benzo[a]hydroxyphenazine-7/8-carboxylic acid; 4C7SFL = potassium 5-carboxy-9-oxo-9H-fluorene-2-sulfonate. Membrane thicknesses are around 150 µm for *s*PIM-SBF membranes.

Supplementary Note 1: Lifetime estimation

Considering the significantly improved chemical/electrochemical stability of redox-active molecules in neutral pH conditions[33,46] and the fact that capacity decay rates correlate well with crossover rates as measured in H-cell dialysis diffusion tests, it can be concluded that the cross-mixing of electrolyte solution is the dominating factor that leads to the battery capacity decay. The lifetime of RFB would be determined by the crossover rates of capacity-limiting redox-active species through the membrane. Here we estimate the lifetime of flow battery stacks based on the overall capacity loss measured in laboratory flow cells at a ferrocyanide concentration of 1 M following the method below, with an assumption that capacity loss solely comes from membrane crossover for ease of calculation.

As demonstrated in this work (Figures 4 and 5), the typical power density of organic redox flow batteries is 1 kW/m^2 (100 mW cm^{-2}) at 0.1 A cm^{-2}. Given a small flow battery cell with an active surface area of 1 m^2 and rated power of 1 kW, and energy storage for 2 h, the energy storage capacity is 2 kWh. The current is equal to the area multiplied by the current density: 0.1 A cm^{-2} × 1 m^2=1000 A; The charge is equal to current multiplied by time: 2 h × 1000 A= 7200 s × 1000 C s^{-1}=7.2 x 10^6 C. Moles of electrons = 7.2×10^6 C / 96,485 C mol^{-1} = 74.6 moles of electrons. Therefore, assuming the energy storage capacity is targeted at 2 kWh for a cell stack with a total active area of 1 m^2, the required battery capacity would be 7.2 × 10^6 C (74.6 mol equivalent electrons) and the charging-discharging duration would be 4 h in total at a current density of 0.1 A cm^{-2}. The materials utilization ratios are 93% and 88% for *s*PIM-SBF-1.40 and Nafion 115, respectively, as measured in laboratory, then 80.2 L and 84.8 L of 1 M ferrocyanide solutions are required to supply 74.6 mol active electrons. The battery lifetime is defined as the duration of battery operation before capacity decay reaches 20% on the condition that the battery only operates one cycle per day and the electrolytes are isolated from the membrane (thus no crossover) when the battery is not in operation.

In flow cells operated at a concentration of 1 M ferrocyanide, RFBs based on *s*PIM-SBF-1.40 and Nafion 115 showed capacity losses of 0.127% per day and 2.66% per day, respectively, which, if we assume capacity decay solely comes from membrane crossover, equals ferrocyanide crossover rates of 3.78 × 10^{-4} mol m^{-2} h^{-1} and 7.92 × 10^{-3} mol m^{-2} h^{-1}, respectively (membrane area is 7 cm^2, ferrocyanide amount is 1 mol L^{-1} × 5 mL = 0.005 mol). If we assume the permeability of ferricyanide is the same as that of ferrocyanide and the concentration difference across the membrane is constant throughout battery service time, the crossover rates of ferro/ferricyanide (*i.e.*, capacity decay rates) in cell stacks at a catholyte concentration of 1 M would also be 3.78 × 10^{-4} mol m^{-2} h^{-1} and 7.92 × 10^{-3} mol m^{-2} h^{-1}, respectively. Additionally, if the battery is assumed to perform one cycle per day with a charging-discharging duration of 4 h, and the electrolytes are isolated from the membrane and stored in external reservoirs during the 20 h when the battery is not in operation, the calendar lifetime of the battery could be amplified by a factor of 6 (24h/4h). Therefore, the estimated lifetime of flow batteries can be derived using the equation (S1):

$$t_{lifetime} = a\frac{C \times 20\%}{PA} \quad \text{(S1)}$$

Where $t_{lifetime}$ is estimated calendar lifetime in h, C is the required number of electrons in mol, P is capacity decay rate in mol m^{-2} h^{-1}, A is the active area of a cell stack in m^2, and a is the calendar time amplification factor. The key parameters are listed in Table S4.

Using *s*PIM-SBF-1.40 as an example, the estimated calendar lifetime is

$$t_{lifetime} = \frac{80.2\ mol \times 20\%}{(3.78 \times 10^{-4}\ mol\ m^{-2}\ h^{-1})}(\frac{24\ h}{4\ h}) = 2.55 \times 10^{5}\ h = 1.06 \times 10^{4}\ day \approx 29.1\ year$$

It should be noted that the above estimation did not consider the degradation of redox species, degradation of membranes, fouling of membranes, pressure of electrolyte solutions, water migrations (Extended data in Supplementary Table 11).

References:

1 Zhang, W., Chen, S., Chen, D. & Ye, Z. Sulfonated binaphthyl-containing poly (arylene ether ketone) s with rigid backbone and excellent film-forming capability for proton exchange membranes. *Polymers* **10**, 1287 (2018).

2 Bae, B., Miyatake, K. & Watanabe, M. Effect of the hydrophobic component on the properties of sulfonated poly (arylene ether sulfone) s. *Macromolecules* **42**, 1873-1880 (2009).

3 Zuo, P. *et al.* Sulfonated microporous polymer membranes with fast and selective ion transport for electrochemical energy conversion and storage. *Angew. Chem. Int. Ed.* (2020).

4 Sorte, E. G. *et al.* Impact of hydration and sulfonation on the morphology and ionic conductivity of sulfonated poly (phenylene) proton exchange membranes. *Macromolecules* **52**, 857-876 (2019).

5 Zhang, C., Kang, S., Ma, X., Xiao, G. & Yan, D. Synthesis and characterization of sulfonated poly (arylene ether phosphine oxide) s with fluorenyl groups by direct polymerization for proton exchange membranes. *J. Membr. Sci.* **329**, 99-105 (2009).

6 Moh, L. C., Goods, J. B., Kim, Y. & Swager, T. M. Free volume enhanced proton exchange membranes from sulfonated triptycene poly (ether ketone). *J. Membr. Sci.* **549**, 236-243 (2018).

7 Jeon, J. W. *et al.* Highly Carboxylate-Functionalized Polymers of Intrinsic Microporosity for CO2-Selective Polymer Membranes. *Macromolecules* **50**, 8019-8027 (2017).

8 Banerjee, S. & Kar, K. K. Impact of degree of sulfonation on microstructure, thermal, thermomechanical and physicochemical properties of sulfonated poly ether ether ketone. *Polymer* **109**, 176-186 (2017).

9 Kusoglu, A. & Weber, A. Z. New Insights into Perfluorinated Sulfonic-Acid Ionomers. *Chem. Rev.* **117**, 987-1104 (2017).

10 Abbott, L. J., Hart, K. E. & Colina, C. M. Polymatic: a generalized simulated polymerization algorithm for amorphous polymers. *Theor. Chem. Acc.* **132**, 1334 (2013).

11 Macrae, C. F. *et al.* Mercury 4.0: from visualization to analysis, design and prediction. *J. Appl. Crystallogr.* **53**, 226-235 (2020).

12 Lide, D. R. *CRC handbook of chemistry and physics*. Vol. 85 (CRC press, 2004).

13 Sing, K. S. W. Reporting physisorption data for gas/solid systems with special reference to the determination of surface area and porosity (Recommendations 1984). *Pure Appl. Chem.* **57**, 603-619 (1985).

14 McKeown, N. B. Polymers of Intrinsic Microporosity (PIMs). *Polymer* **202**, 122736 (2020).

15 Nightingale Jr, E. R. Phenomenological theory of ion solvation. Effective radii of hydrated ions. *The Journal of Physical Chemistry* **63**, 1381-1387 (1959).

16 Park, M. *et al.* A High Voltage Aqueous Zinc–Organic Hybrid Flow Battery. *Advanced Energy Materials* **9**, 1900694 (2019).

17 Zhang, C. *et al.* Phenothiazine-Based Organic Catholyte for High-Capacity and Long-Life Aqueous Redox Flow Batteries. *Adv. Mater. (Weinheim, Ger.)* **31**, 1901052 (2019).

18 Chen, Q., Eisenach, L. & Aziz, M. J. Cycling Analysis of a Quinone-Bromide Redox Flow Battery. *J. Electrochem. Soc.* **163**, A5057-A5063 (2015).

19 Gerhardt, M. R. *et al.* Anthraquinone Derivatives in Aqueous Flow Batteries. *Advanced Energy Materials* **7**, 1601488 (2017).

20 Jin, S. *et al.* A Water-Miscible Quinone Flow Battery with High Volumetric Capacity and Energy Density. *ACS Energy Letters* **4**, 1342-1348 (2019).
21 Janoschka, T. *et al.* An aqueous, polymer-based redox-flow battery using non-corrosive, safe, and low-cost materials. *Nature* **527**, 78-81 (2015).
22 Liu, T., Wei, X., Nie, Z., Sprenkle, V. & Wang, W. A total organic aqueous redox flow battery employing a low cost and sustainable methyl viologen anolyte and 4 ‐ HO ‐ TEMPO catholyte. *Advanced Energy Materials* **6**, 1501449 (2016).
23 Janoschka, T., Martin, N., Hager, M. D. & Schubert, U. S. An aqueous redox ‐ flow battery with high capacity and power: the TEMPTMA/MV system. *Angew. Chem. Int. Ed.* **55**, 14427-14430 (2016).
24 Hu, B. *et al.* Improved radical stability of viologen anolytes in aqueous organic redox flow batteries. *Chem. Commun.* **54**, 6871-6874 (2018).
25 Luo, J., Hu, B., Debruler, C. & Liu, T. L. A π ‐ conjugation extended viologen as a two ‐ electron storage anolyte for total organic aqueous redox flow batteries. *Angew. Chem. Int. Ed.* **57**, 231-235 (2018).
26 Liu, Y. *et al.* A Long-Lifetime All-Organic Aqueous Flow Battery Utilizing TMAP-TEMPO Radical. *Chem* **5**, 1861-1870 (2019).
27 Beh, E. S. *et al.* A neutral pH aqueous organic–organometallic redox flow battery with extremely high capacity retention. *ACS Energy Letters* **2**, 639-644 (2017).
28 DeBruler, C. *et al.* Designer two-electron storage viologen anolyte materials for neutral aqueous organic redox flow batteries. *Chem* **3**, 961-978 (2017).
29 Hu, B., DeBruler, C., Rhodes, Z. & Liu, T. L. Long-cycling aqueous organic redox flow battery (AORFB) toward sustainable and safe energy storage. *J. Am. Chem. Soc.* **139**, 1207-1214 (2017).
30 Huang, J. *et al.* Spatially Constrained Organic Diquat Anolyte for Stable Aqueous Flow Batteries. *ACS Energy Letters* **3**, 2533-2538 (2018).
31 Luo, J. *et al.* Unprecedented Capacity and Stability of Ammonium Ferrocyanide Catholyte in pH Neutral Aqueous Redox Flow Batteries. *Joule* **3**, 149-163 (2019).
32 Liu, Y. *et al.* Screening Viologen Derivatives for Neutral Aqueous Organic Redox Flow Batteries. *ChemSusChem* **13**, 2245-2249 (2020).
33 Ji, Y. *et al.* A Phosphonate ‐ Functionalized Quinone Redox Flow Battery at Near ‐ Neutral pH with Record Capacity Retention Rate. *Advanced Energy Materials* **9**, 1900039 (2019).
34 Jin, S. *et al.* Near Neutral pH Redox Flow Battery with Low Permeability and Long ‐ Lifetime Phosphonated Viologen Active Species. *Advanced Energy Materials* **10**, 2000100 (2020).
35 Kwabi, D. G. *et al.* Alkaline quinone flow battery with long lifetime at pH 12. *Joule* **2**, 1894-1906 (2018).
36 Orita, A., Verde, M. G., Sakai, M. & Meng, Y. S. A biomimetic redox flow battery based on flavin mononucleotide. *Nature communications* **7**, 1-8 (2016).
37 Hollas, A. *et al.* A biomimetic high-capacity phenazine-based anolyte for aqueous organic redox flow batteries. *Nature Energy* **3**, 508-514 (2018).
38 Lin, K. *et al.* A redox-flow battery with an alloxazine-based organic electrolyte. *Nature Energy* **1**, 1-8 (2016).
39 Lin, K. *et al.* Alkaline quinone flow battery. *Science* **349**, 1529-1532 (2015).

40 Yang, Z. *et al.* Alkaline Benzoquinone Aqueous Flow Battery for Large-Scale Storage of Electrical Energy. *Advanced Energy Materials* **8**, 1702056 (2018).
41 Sun, P. *et al.* 110th Anniversary: Unleashing the Full Potential of Quinones for High Performance Aqueous Organic Flow Battery. *Ind. Eng. Chem. Res.* **58**, 3994-3999 (2019).
42 Tong, L. *et al.* Molecular Engineering of an Alkaline Naphthoquinone Flow Battery. *ACS Energy Letters* **4**, 1880-1887 (2019).
43 Wu, M. *et al.* Extremely Stable Anthraquinone Negolytes Synthesized from Common Precursors. *Chem* **6**, 1432-1442 (2020).
44 Wang, C. *et al.* Molecular Design of Fused-Ring Phenazine Derivatives for Long-Cycling Alkaline Redox Flow Batteries. *ACS Energy Letters* **5**, 411-417 (2020).
45 Feng, R. *et al.* Reversible ketone hydrogenation and dehydrogenation for aqueous organic redox flow batteries. *Science* **372**, 836 (2021).
46 Kwabi, D. G. *et al.* Alkaline quinone flow battery with long lifetime at pH 12. *Joule* **2**, 1894-1906 (2018).
